# Early and mid-gestation Zika virus (ZIKV) infection in the olive baboon (*Papio anubis*) leads to fetal CNS pathology by term gestation

**DOI:** 10.1371/journal.ppat.1010386

**Published:** 2022-08-15

**Authors:** Sunam Gurung, Darlene Reuter, Abby Norris, Molly Dubois, Marta Maxted, Krista Singleton, Marisol Castillo-Castrejon, James F. Papin, Dean A. Myers

**Affiliations:** 1 Department of Obstetrics and Gynecology, University of Oklahoma Health Sciences Center, Oklahoma City, United States of America; 2 Division of Comparative Medicine, University of Oklahoma Health Sciences Center, Oklahoma City, United States of America; 3 Department of Pathology, University of Oklahoma Health Sciences Center, Oklahoma City, United States of America; University of Minnesota Twin Cities, UNITED STATES

## Abstract

Zika virus (ZIKV) infection in pregnancy can produce catastrophic teratogenic damage to the developing fetus including microcephaly and congenital Zika syndrome (CZS). We previously described fetal CNS pathology occurring by three weeks post-ZIKV inoculation in Olive baboons at mid-gestation, including neuroinflammation, loss of radial glia (RG), RG fibers, neuroprogenitor cells (NPCs) resulting in disrupted NPC migration. In the present study, we explored fetal brain pathologies at term gestation resulting from ZIKV exposure during either first or second trimester in the Olive baboon. In all dams, vRNA in whole blood resolved after 7 days post inoculation (dpi). One first trimester infected dam aborted at 5 dpi. All dams developed IgM and IgG response to ZIKV with ZIKV IgG detected in fetal serum. Placental pathology and inflammation were observed including disruption of syncytiotrophoblast layers, delayed villous maturation, partially or fully thrombosed vessels, calcium mineralization and fibrin deposits. In the uterus, ZIKV was detected in ¾ first trimester but not in second trimester infected dams. While ZIKV was not detected in any fetal tissue at term, all fetuses exhibited varying degrees of neuropathology. Fetal brains from ZIKV inoculated dams exhibited a range of gross brain pathologies including irregularities of the major gyri and sulci of the cerebral cortex and cerebellar pathology. Frontal cortices of ZIKV fetuses showed a general disorganization of the six-layered cortex with degree of disorganization varying among the fetuses from the two groups. Frontal cortices from ZIKV inoculation in the first but not second trimester exhibited increased microglia, and in both trimester ZIKV inoculation, increased astrocyte numbers (white matter). In the cerebellum, increased microglia were observed in fetuses from both first and second trimester inoculation. In first trimester ZIKV inoculation, decreased oligodendrocyte precursor cell populations were observed in fetal cerebellar white matter. In general, our observations are in accordance with those described in human ZIKV infected fetuses.

## Author summary

Infection of pregnant women with Zika virus can cause severe birth defects and disabilities in the developing fetus with microcephaly as the characteristic zika specific neuropathology. Being natural hosts for this virus, non-human primates (NHPs) have provided excellent models to understand the origins of fetal brain damage induced by Zika virus. In a previous study using the baboon as a non-human primate model for Zika virus infection, we observed neuronal precursor and glial cells targeting by Zika virus in the fetal brain during the early stages after inoculation of the pregnant baboons. In this study, we infected pregnant baboons in either the first or second trimesters with Zika virus and examined fetal brains at the end of pregnancy. Fetuses from the infected dams exhibited a range of visible brain pathology such as defects in brain folding and cerebellar damage. In the frontal cortex, fetuses showed a general neuronal disorganization in the cortex in both first and second trimester infected pregnancies, with a wide variation in the severity of defects noted. Fetuses from first trimester infected baboon mothers showed greater damage compared to the second trimester infected baboons. In general, our observations are in accordance with those described in humans infected with Zika virus.

## Introduction

The epidemic potential and transmission of emerging and re-emerging flaviviral diseases such as Zika virus (ZIKV) pose a major threat to the global public health and economy [1,2]. While ZIKV infections have declined since peaking in ~2017, where it had spread to 84 countries with over 800,000 cases reported in the Americas alone (PAHO), it remains a global threat due to continued presence in tropical American regions, lack of a vaccine, and continued expansion of its primary mosquito vector, *Aedes aegypti*. Although ZIKV infection is typically mild or asymptomatic, ZIKV can exert major pathologies during pregnancy including spontaneous abortion, intrauterine fetal death, fetal growth restriction, neurological damage and microcephaly. Collectively these effects on the fetus are termed congenital zika syndrome (CZS) [3,4]. Newborns of women infected with ZIKV during pregnancy have a 5–14% risk of developing CZS [5] [6].

Studies show poor developmental prognosis for children with CZS including severe developmental injuries by 30 months of age [7] and below average neurodevelopmental assessment (cognitive, language, motor, autism spectral disorder) between 7 and 32 months of age [8]. Neurological impairment, including development of microcephaly in infants born with normal head circumference, have also been reported in infants born without clinical abnormalities [9,10] [11,12]. In the USA, 9% of children born to ZIKV infected mothers were reported to have at least one neurodevelopmental abnormality prior to 2 years of age [13]. These data collectively imply that fetal neurological damage induced by ZIKV *in utero* is more common than initially thought.

The mechanisms driving neurological damage to the developing human fetus from a ZIKV infected mother have not been fully elucidated. Two primary animal models have been employed to study mechanisms of ZIKV induced fetal neuropathology, mice and non-human primates (NHPs). Mouse models have limitations due to not being a natural host for ZIKV thus necessitating the use of interferon null mice or direct viral injection into the developing fetus as well as considerable differences in neurodevelopment compared to primates [14–17]. Being natural hosts for ZIKV and related flaviviruses, NHPs have proven excellent translational models for studying ZIKV pathogenesis [18,19]. Zika virus infection has been extensively studied in baboons [20–23], macaques (rhesus [24–30], pigtail [31,32], cynomolgus [33, 34]), marmosets and tamarins (*Callithrix jacchus; Saguinas labiatus* [35,36]*)*. Similar to humans, intrauterine fetal death and/or miscarriage has been reported as a common outcome (26%) following ZIKV infection in macaques and baboons [37]. While microcephaly has not been reported in ZIKV infected macaques, a range of fetal or infant neuropathological outcomes have been described from none [24,25] to notable [28,31], including CNS lesions (microcalcifications, hemorrhage, vasculitis, necrosis), gliosis (microglia, astrocytes), white matter injury, calcification, loss of NPCs, reduced brain size and agyria, with the latter being rare [28,31,32,38]. A range of placental pathology has been described in macaques from negligible [25,31] to severe, including placental insufficiency [26,28]. Hypoxia and inflammation in the developing fetus due to placental/vascular insufficiency may contribute to fetal brain damage and CZS [28]. ZIKV infection earlier in pregnancy is associated with more severe fetal brain pathology and CZS in macaques consistent with human data [39]. A number of studies in rhesus macaques reported a high rate of vertical transfer of ZIKV, ranging from 80–90% [26,28,29,40] to 100% vertical transmission rates [25,41], while two studies did not observe vertical transmission [42,43]. In humans, the rate of vertical transfer has been estimated between 10–35% [44,45].

We developed an olive baboon (*Papio anubis*) model to study ZIKV pathogenesis. The olive baboon is similar to humans in terms of size, genetics, placentation, gestation, brain development and immune repertoire making the baboon an excellent translational NHP model to study ZIKV infection [46–48]. The baboon is permissive to flavivirus infection and replication, including ZIKV [48,49]. In a previous study, we focused on the initial three weeks post-subcutaneous ZIKV inoculation (French Polynesian strain H/PF/2013) during mid-gestation to decipher the timing of vertical transfer and early events of fetal CNS targeting [21]. Prior studies in macaques typically examined outcomes at term gestation or early post-delivery. We observed vertical transfer in ½ of the infected dams. In one fetus at three weeks post-maternal inoculation, we observed extensive neuropathology including a significant loss in radial glia (RG), RG fibers, decreased NPCs, astrogliosis and increased reactive microglia as well as decreased oligodendrocyte precursor cells (OPCs). Consistent with the loss of RG fibers, which serve as scaffolding for neuronal and NPC migration to the cortical plate, we noted cortical plate neuronal disorganization of the characteristic six-layered cortex. To our knowledge, we were the first to report the loss of RG fibers due to ZIKV infection in primates, which had been previously seen in microcephalic human fetuses and mouse models [21]. The present study extended these findings by examining fetal neurological outcome at term gestation following ZIKV inoculation during late first trimester or at mid-gestation in the olive baboon.

Herein, we describe inoculation of four early-gestation and three mid-gestion timed-pregnant olive baboons with the contemporary Puerto Rican ZIKV strain (PRVABC59, 2015). We report extensive fetal brain pathology from early and mid-gestation infected dams, one fetal abortion in the early gestation group, viral persistence of ZIKV RNA in the uterus of early gestation infected dams and in various lymph nodes in both the early and mid-gestation infected dams at the time of termination of the study.

## Results

### Maternal ZIKV infection, observed clinical signs and pregnancy outcome

Adult timed pregnant female olive baboons (early gest n = 4 Dams 1-4E; mid-gest n = 3, Dams 1-3M) were infected subcutaneously with 10^4^ plaque forming units of the Puerto Rican ZIKV isolate (PRVABC59). The sampling procedure for each dam is detailed in **Table 1**.

**Table 1:** Study design and timeline.

Days Post-Inoculation (PR Strain 10^4 pfu/ml)									
	ID	0	4	7	14	21	42	73/80	115
**Early-Gestation Inoculation**	**Dam 1E (56–172 DG)**	X	X	X	X	X	X	X	X **N**
	**Dam 2E (58–169 DG)**	X	X	X	X	X	X	X	X **N**
	**Dam 3E (55–169 DG)**	X	X	X	X	X	X	X	X **N**
	**Dam 4E (55–59 DG)**	X	X **N, SA**						
**Mid-Gestation Inoculation**	**Dam 1M (92–167 DG)**	X	X	X	X	X	X	X **N**	
	**Dam 2M (94–169 DG)**	X	X	X	X	X	X	X **N**	
	**Dam 3M (97–167 DG)**	X	X	X	X	X	X	X **N**	

X: whole blood, saliva and vaginal swab sample collection days; N: Necropsy; SA: Spontaneous abortion

Clinical findings and pregnancy outcomes are detailed in **Table 2**. None of the dams exhibited body temperatures (obtained under ketamine sedation) greater than 1°C above day 0 over the course of the study.

**Table 2:** Summary of maternal clinical findings and pregnancy outcome.

ID (Inoculation-Necropsy) DG	D0	D4	D7	D14	D21	D40/D42	D73/D80	D115	Fetus
DAM1E (56–172) DG	N/A	None	Slight erythema (inguinal, axillary, injection site)	Mild papular rash (axillary), moderate papular rash (inguinal), slight erythema on chest and abdomen	Mild papular rash (axillary), severe papular rash (inguinal)	Mild papular rash (axillary, inguinal)	Moderate to severe papular rash (axillary), slight papular rash (inguinal)	None	V
DAM2E (58–169) DG	N/A	None	None	Slight erythema (inguinal)	None	None	None	None	V
DAM3E (55–169) DG	N/A	None	None	None	None		Slight erythema (inguinal)	None	V
DAM4E (55–59) DG	N/A	None							SA
DAM1M (92–167) DG	Slight erythema (axillary, inguinal) before inoculation	Slight erythema (axillary, inguinal)	Slight erythema (axillary, inguinal and injection site), localized redness on the upper abdomen and chest, significant conjunctivitis	Slight erythema (injection site), localized redness on the upper abdomen and chest, mild conjunctivitis	Slight erythema (axillary, inguinal, chest injection site), slight conjunctivitis	Slight erythema (inguinal)	None		V
DAM2M (94–169) DG	N/A	Slight erythema (axillary and inguinal)	Mild conjunctivitis	Slight conjunctivitis	Slight erythema (inguinal)	None	None		V
DAM3M (97–167) DG	Moderate dermatitis (inguinal) prior to inoculation	Slight erythema (injection site)	Slight erythema (axillary), mild conjunctivitis	Slight erythema (axillary), slight conjunctivitis	None	None	None		V

DG, gestation day age; N/A, not applicable; V, Viable; SA, Spontaneous abortion; E, early-gestation inoculation group; M, mid-gestation inoculation group

### Maternal ZIKV viral loads

**Fig 1:**
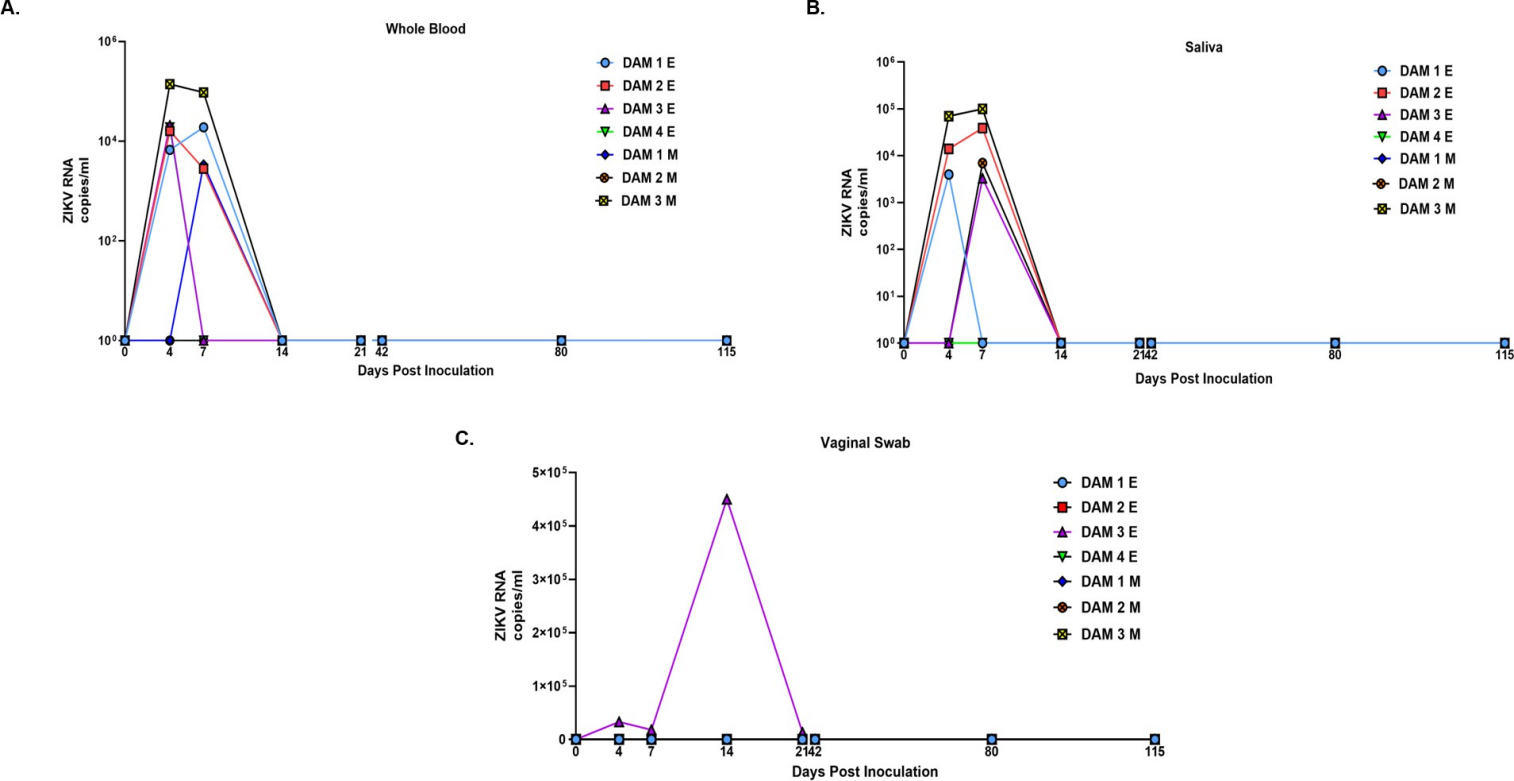
ZIKV RNA (copies per milliliter) in whole blood (**A**), saliva (**B**) and vaginal swab (**C**) samples in baboons inoculated subcutaneously with PR ZIKV isolate during early and mid-gestation. ZIKV RNA was prepared from samples collected from each animal at the indicated days post inoculation and quantitated by one-step qRT-PCR.

In the early-gestation cohort, ZIKV RNAemia initiated at 4 dpi in Dams 1E, 2E, 3E and 4E. Dams 1E and 2E were also RNAemic at 7 dpi but not at later time points. ZIKV RNA was detected in saliva from Dams 1E and 3E at 4 dpi (Dam 1E), 4, 7 dpi (Dam 2E) and 7 dpi (Dam 3E) (**Fig 1**). We did not detect ZIKV RNA in vaginal swabs obtained at any time point for Dams 1E and 2E. ZIKV RNA was detected in vaginal swabs from Dam 3E at 4, 7, 14 and 21 dpi. Dam 4E aborted 5 dpi, therefore, the final samples for this dam were collected during necropsy on day 5 post inoculation.

In the mid-gestation cohort, RNAemia was observed at 7 dpi for Dam 1M, and at 4 and 7 dpi for Dam 3M. Dam 2M did not have detectable RNAemia at any time point. ZIKV RNA was detected in saliva samples from Dam 2M on 7 dpi and Dam 3M at 4 and 7 dpi. Dam 1M did not have detectable ZIKV RNA in saliva at any time point. None of the dams had detectable ZIKV RNA in the vaginal swabs collected at any time point.

Reproductive tissues (vagina, cervix, uterus, ovaries) and lymph nodes (LNs) (axial, inguinal, mesenteric) were examined for ZIKV RNA from the dams at the time of necropsy (**Table 3**). In the early-gestation cohort, of the four uterine sites sampled per dam, ZIKV RNA was detected in 1/4 in Dam 1E, 2/4 in 3E and in 3/4 samples in Dam 4E. Inguinal LN from Dam 1E was positive for ZIKV RNA and Inguinal and Axial LNs from Dam 4E were positive for ZIKV RNA. No ZIKV RNA was detected in ovaries, cervix or vaginal tissues for the early gestation dams.

**Table 3:** ZIKV RNA in select maternal, fetal tissues (copies per mg tissue or per ml of fluid).

Fluids/Tissues	Dam 1E (Early)	Dam 2E (Early)	Dam 3E (Early)	Dam 4E (Early)	Dam 1M (Mid)	Dam 2M (Mid)	Dam 3M (Mid)	Control (n = 3)
Gestation day start/end	56–172 dG	58–169 dG	55–169 dG	55–59 dG	92–167 dG	94–169 dG	97–167 dG	169, 171, 166 dG
**Maternal Samples**								
Uterus	10^4	<LD	10^6	10^6	<LD	<LD	<LD	-
Lymph node	10^3	<LD	<LD	10^5	10^4	10^3	10^4	-
**Fetal Samples**								
Fetal weight	1.06 kg	0.665 kg	1.06 kg	N/A	0.885 kg	0.965 kg	0.780 kg	0.80, 1.05, 0.8 kg
Viability	V	V	V	Aborted	V	V	V	V
Sex	F	F	M	-	M	F	F	M, M, F
Placenta	<LD	<LD	<LD	-	<LD	<LD	<LD	-
Cord Blood	<LD	<LD	<LD	-	<LD	<LD	<LD	-
Amniotic Fluid	<LD	<LD	<LD	-	<LD	<LD	<LD	-
Tissues	<LD	<LD	<LD	-	<LD	<LD	<LD	-

dG, gestation day age; <LD below level of detection (cutoff was 1x10^2^); Fetal tissues tested for ZIKV RNA were cortex (4 lobes), cerebellum, hippocampus, lung, liver, spleen, gonad, stomach, intestine, eye and thymus.

None of the reproductive tissues collected from the mid-gestation cohort had detectable ZIKV RNA. Axial and Inguinal LNs from Dam 1M, Axial LNs from Dam 2M and 3M had detectable ZIKV RNA.

### Placental, amniotic fluid and fetal ZIKV viral loads

Fetuses are coded to match the dams (e.g., Dam 1 = Fetus 1). Placenta, fetal tissues (frontal lobe, occipital lobe, parietal lobe, temporal lobe, cerebellum, hippocampus, lung, liver, spleen, gonad, stomach, intestine, eye and thymus) and amniotic fluid collected at the time of necropsy from early and mid-gestation animals did not have detectable ZIKV RNA (**Table 3**).

### ZIKV specific IgM and IgG

#### IgM

**Fig 2:**
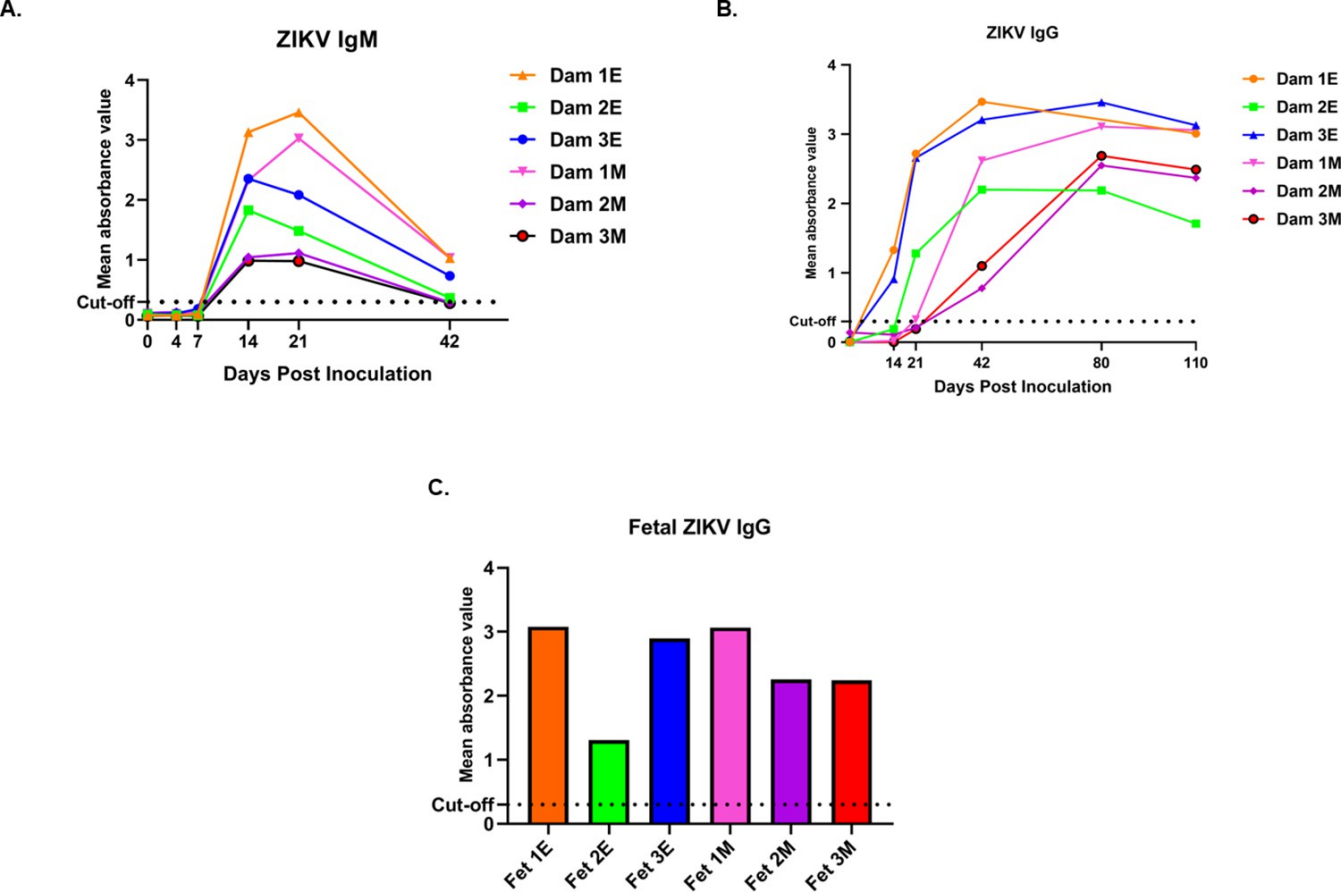
Detection of anti-ZIKV antibody responses in pregnant baboon serum. Antibodies against ZIKV was determined by ELISA for maternal IgM (**A**) and IgG (**B**) and fetal IgG (**C**). anti-ZIKV IgM were detected at 14 days post-inoculation in all six baboons sampled at this time point. In the early-gestation group, dams 1E and 3E had ZIKV IgG at 14 dpi and dam 2E had at 21 dpi. In the mid-gestation inoculation group, all three dams had IgG by 21dpi. All six fetuses had anti-ZIKV IgG at levels similar to the mothers indicating efficient FcγR transfer of IgG across the placenta. Mean absorbance value was calculated from duplicates for each sample. Absorbance for all serum samples was read at 450nm for IgM and 405nm for ZIKV IgG assays. Cut off values for both assays were set per manufacturer’s criteria for each assay.

All dams were negative for ZIKV IgM prior to inoculation and remained negative through 7 dpi. Dams 1E, 2E and 3E from the early-gest cohort and Dams 1M and 2M from the mid-gestation cohort had IgM response from 14 through 42 dpi. Dam 3M had ZIKV IgM on 14 and 21 dpi and had the weakest IgM response compared to all other dams. Dams 1E and 3E from early-gestation and 1M from mid-gestation group exhibited the most robust IgM response (**Fig 2A**). ZIKV IgM was not detected in fetal cord blood collected at necropsy of any fetus (Fet 1E, 2E, 3E, Fet 1M, 2M and 3M).

#### IgG

No dams had serum ZIKV IgG by 7 dpi. In the early-gestation cohort, Dams 1E and 3E had ZIKV IgG from14 dpi, gradually increasing to 115 dpi. Dam 2E had ZIKV IgG from 21–110 dpi. In the mid-gestation cohort, Dam 1M had increasing ZIKV IgG from 14 to 73 dpi and Dams 2M and 3M had ZIKV IgG from 21 to 73 dpi. ZIKV IgG was also detected in the cord blood of all fetuses from early and mid-gestation dams (**Fig 2B and 2C**).

### CNS Histology and immunohistochemistry

#### Brain pathology

**Fig 3:**
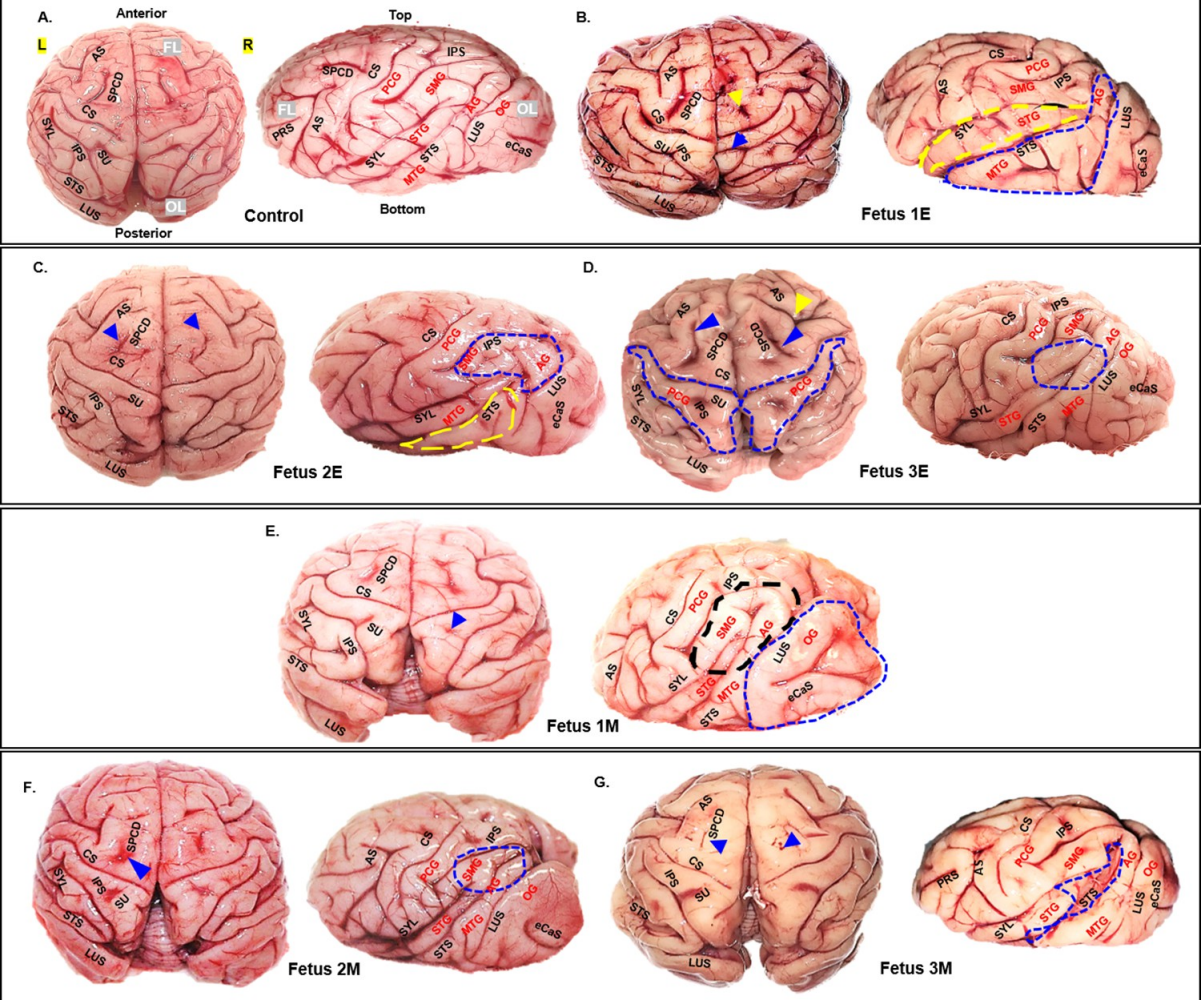
Gyri/sulci malformations in brains from fetuses form early and mid-gestation ZIKV inoculated dams compared to controls (**A**). Top and side view of fetal brains showing major gyri and sulci formations. Abnormal sulci (abnormal sulcal length and depth, asymmetry across cerebral hemispheres) or missing sulci are marked by arrows. Gyri malformations (enlargement, asymmetric across left and right cerebral hemispheres) are highlighted by dotted areas compared to the control fetal brain (**A**). Fetuses from early gestation inoculated dams **(B, C, D)**; fetuses from mid-gestation inoculated dams (**E, F, G**). Frontal Lobe (FL), Occipital Lobe (OL), Parietal Lobe (PL), Left Cerebral Hemisphere (L), Right Cerebral Hemisphere (R), Arcuate Sulcus (ARS), Central Sulcus (CS), External calcarine Sulcus (eCaS), Inferior Parietal Sulcus (IPS), Lunate Sulcus (LUS), Superior Pre-central dimple (SPCD), Superior Post-central dimple (SU), Principal Sulcus (PRS), Superior Temporal Sulcus (STS), Sylvian Fissure/Lateral Sulcus (SYL), Postcentral Gyrus (PCG), Supramarginal Gyrus (SMG), Superior Temporal Gyrus (STG), Angular Gyrus (AG), Middle Temporal Gyrus (MTG), Occipital Gyrus (OG).

**Fig 4:**
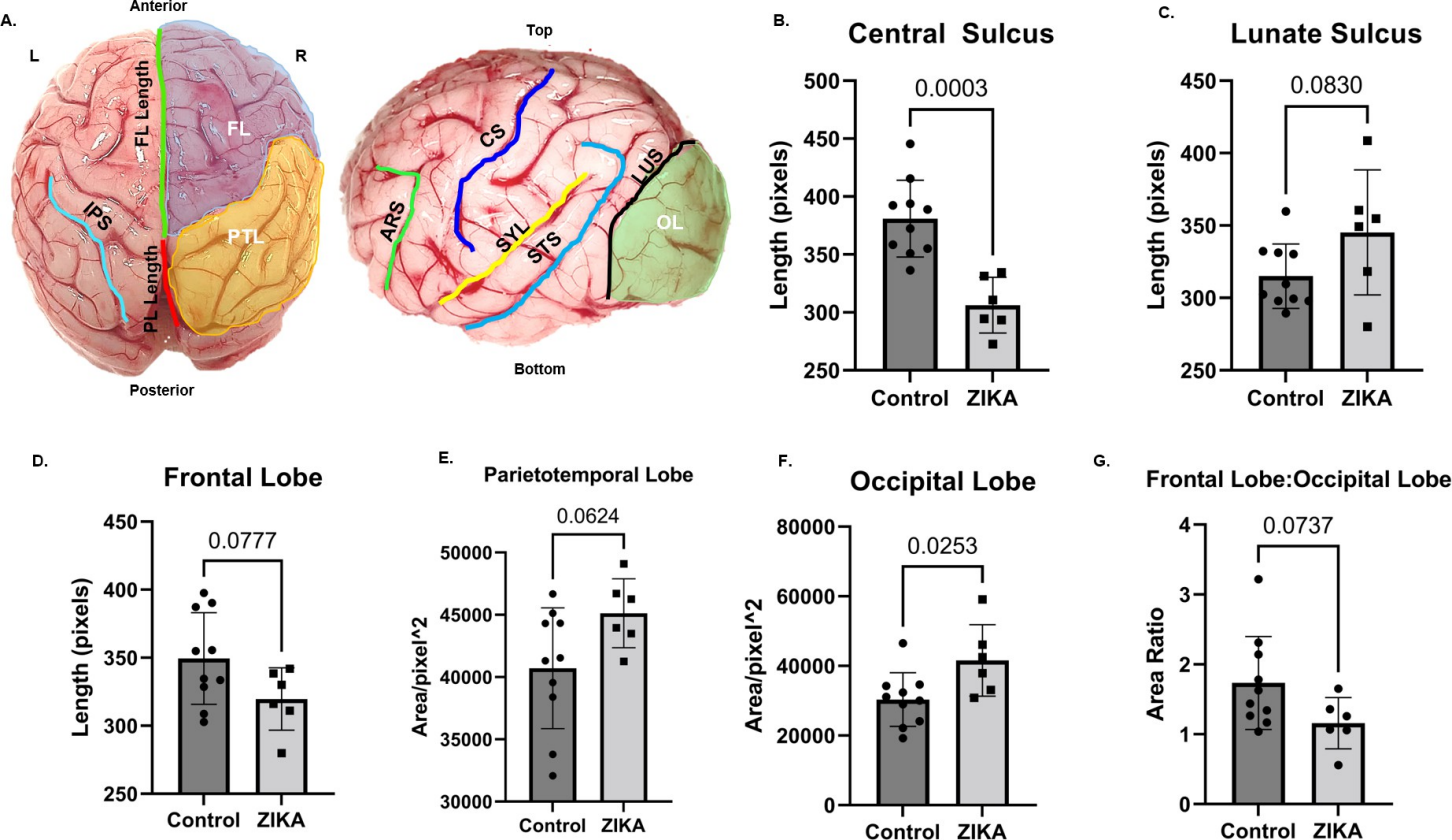
Differences in sulcal length and cerebral lobe surface area in brains from fetuses from early and mid-gestation ZIKV inoculated dams compared to controls. Image showing lengths of different sulcis and surface areas of different cerebral lobes measured using Image J (**A**). Measurements of CS, ARS, SYL, STS, LUS length and OL surface area were done on left hemisphere image (side view) of each brain. Measurements of right and left IPS length, FL and PTL surface areas were done on top view image of the brain. FL length was measured by drawing a line from tip of the frontal lobe to CS (**A**, top view, green line) and PL length was measured by drawing a line from CS to LUS (**A**, top view, red line). Significant decrease in length of central sulcus (**B**) and increase in surface area of Occipital lobe (**F**) was seen in fetal brains from ZIKV inoculated dams compared to control brains (n = 10). In fetal brains from ZIKV inoculated dams, a trend towards reduction in frontal lobe length (**D**), in frontal lobe to occipital lobe area ratio (**G**) and increase in length of lunate sulcus (**C**), and in surface area of parieto-temporal lobe surface area (**E**) was seen compared to control brains. (Error bars depict SD, unpaired *t*-test, p<0.05 considered significant). Frontal Lobe (FL), Occipital Lobe (OL), Parietal Lobe (PL), Parieto Temporal area (PTL), Left Cerebral Hemisphere (L), Right Cerebral Hemisphere (R), Arcuate Sulcus (ARS), Central Sulcus (CS), Inferior Parietal Sulcus (IPS), Lunate Sulcus (LUS), Superior Temporal Sulcus (STS), Sylvian Fissure/Lateral Sulcus (SYL).

Post necropsy, ZIKV exposed fetal brains were visually assessed for gross structural defects, primarily, overall size, and gyri and sulci formation compared to the control brains (**Fig 3**). This comparative visual assessment was made based on previous neuroanatomic publications detailing gyri and sulci formation in fetal baboons [50] macaques [51] multiple non-human primates (old and new world monkeys) [52] and the brain atlas of rhesus and cynomolgus macaques [53]. **Fig 3A** shows a representative control fetal brain with different major sulci and gyri labeled. Fetal brains from ZIKV exposed dams (**Fig 3B–3G**) exhibited a range of irregularities of the major gyri and sulci in the different lobes of the cerebral cortex regardless of gestation age at inoculation as described in **Table 4**. Superficial measurements of major sulcal lengths and surface areas of cerebral lobes (**Fig 4A**) showed significant reduction in central sulcal length (**Fig 4B**) in ZIKV fetuses and a trend towards increase in length of lunate sulcus (**Fig 4C**) compared to control fetal brains. Our measurements also showed a trend towards reduction in frontal lobe length (**Fig 4D**), increase in parieto-temporal lobe surface area (**Fig 4E**) and a significant increase in occipital lobe surface area (**Fig 4F**) in ZIKV brains than control brains. The frontal lobe to occipital lobe area ratio was higher in control brains than in ZIKV brains (**Fig 4G**) suggesting differential effect of ZIKV infection in cerebral lobe development with frontal and occipital lobe showing changes due to ZIKV infection.

**Table 4:** Gyri and Sulci malformations in ZIKV fetal brains.

Animal ID	Sulci	Gyri
**Fet 1E**	Asymmetric SPCD on left and right hemisphere (yellow arrow, Fig 3B top view). Asymmetric SU on left and right hemisphere, blue arrow marks unusual bifurcation of SU seen on right hemisphere. SU on left hemisphere appears diminutive compared to control (Fig 3B top view)	STG (yellow highlighted area) and MTG (blue highlighted area) appear enlarged compared to control brain (Fig 3B side view).
**Fet 2E**	SPCD on the right hemisphere appears convex shaped similar to the left side as opposed to the concave shape seen in the control brain (blue arrow, Fig 3C top view)	SMG appears reduced in size whereas AG appears enlarged (blue highlighted area). MTG appears reduced in size due to decrease in STS length (yellow highlighted area, Fig 3C side view).
**Fet 3E**	Changes in sulcal depth of sulcis SPCD (blue arrows), AS (yellow arrow) and SU (blue highlighted area) seen in this brain compared to the control brain (Fig 3D top view). SYL and STS appear to merge which is not seen in the control brain (blue highlighted area, Fig 3D side view).	PCG (blue highlighted area) appears enlarged (Fig 3D top view)
**Fet 1M**	SU is missing in the right hemisphere (blue arrow, Fig 3E top view).	SMG appears enlarged whereas STG appears reduced in size (black highlighted area, side view). OG appears much enlarged than the control brain (blue highlighted area, Fig 3E side view).
**Fet 2M**	SPCD on the left hemisphere appears diminutive compared to the control (blue arrow, Fig 3F top view).	SMG, STG and MTG appear reduced in size compared to control (blue highlighted area, Fig 3F side view).
**Fet 3M**	SPCD appears diminutive compared to control (blue arrows. STS appear abnormally deep and flaccid (blue highlighted area, Fig 3G side view).	MTG and OG appear reduced in size compared to the control brain (Fig 3G side view).

AG, Angular gyrus; AS, Arcuate sulcus; MTG, Middle Temporal Gyrus; OG, Occipital gyrus; PCG, Postcentral gyrus; SMG, Supramarginal gyrus; SPCD, Superior Precentral dimple; STG, Superior Temporal gyrus; STS, Superior Temporal Sulcus; SU, Superior Postcentral dimple; SYL, Sylvian fissure.

### Histology of frontal cortex and cerebellum

**Fig 5:**
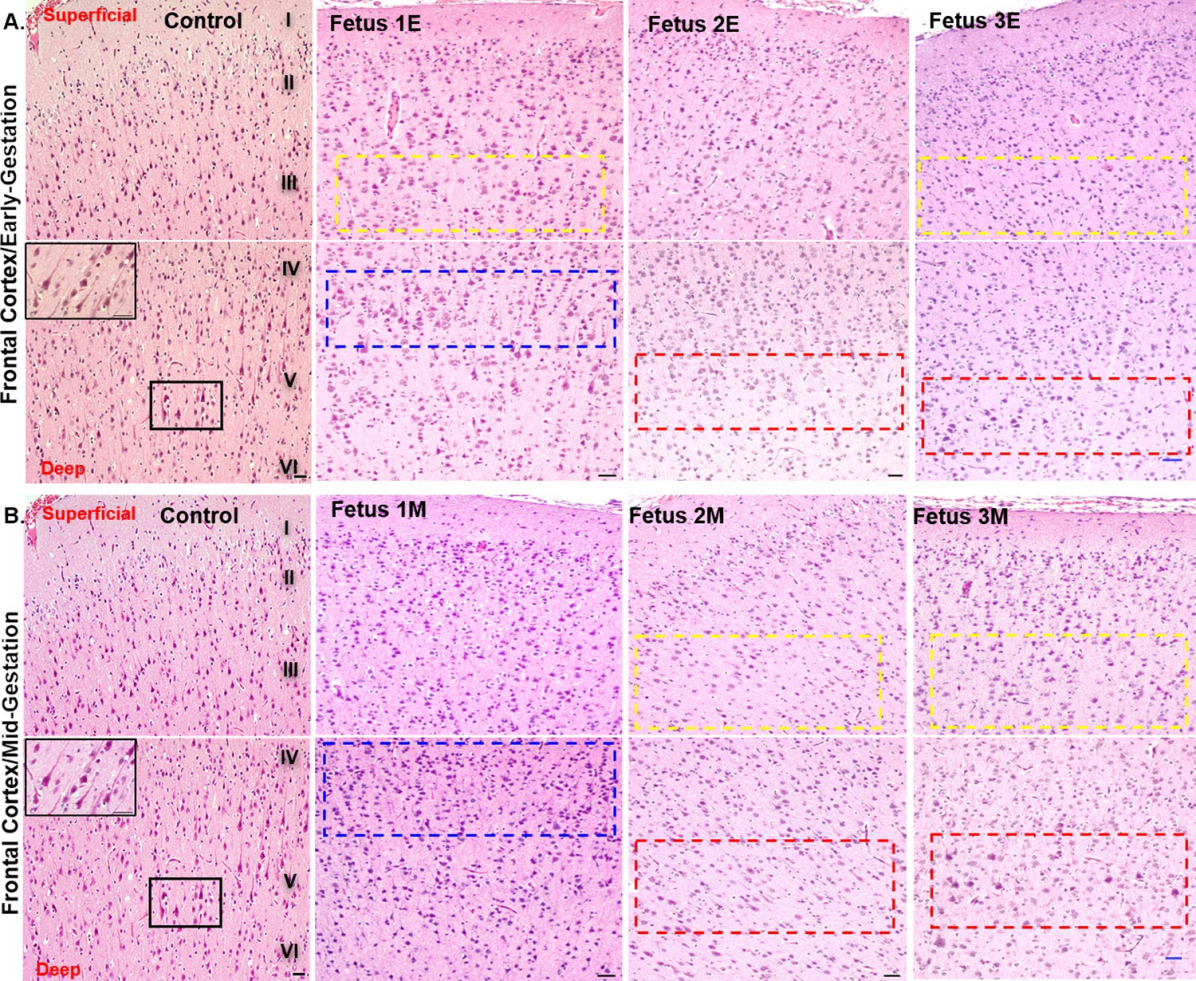
ZIKV inoculation disrupts formation of six-layered cortex in a developing fetal brain. Representative images from H&E staining of control and early-gestation (**A**) and mid-gestation (**B**) cortical plate of the fetal frontal cortex. The histological stain shows control fetal brain with orderly formation of six layers of cortex compared to the disturbance of this process in the fetal cortical plates from fetuses from early (**A**) and mid-gestation (**B**) inoculated dams. Yellow dotted areas highlight lack of small pyramidal neurons in Layer III. The blue dotted areas highlight presence of small pyramidal neurons normally present in Layer III seen instead shifted down to Layer IV which normally consists mostly of stellate cells and smaller portion of pyramidal cells. The red dotted areas highlight lack of large pyramidal neurons in Layer V of the cortical plate as seen in the control image (box and inset). In Fetus 3E and 3M, some of the cells highlighted in the red dotted areas could be cells other than the large pyramidal cells typical of Layer V since these cells appear lacking the characteristic apical dendrites emerging from the soma of the pyramidal neurons as seen in the control brain (inset). (Scale bar = 100μm).

H&E staining of the frontal cortices of ZIKV fetuses showed a general disorganization of the six-layered cortex in early (1E, 2E, 3E) and mid (1M, 2M, 3M) gestation compared to the control fetal brains. The category and degree of disorganization of the fetal cortex in different layers of the cortex varied among the fetuses (**Fig 5A and 5B**). In Fet 1E, a lack of small pyramidal neuronal population in Layer III was observed as highlighted by the yellow box compared to the control. Instead, small and large pyramidal neurons were seen in Layer IV which normally consists of mostly stellate cells and some pyramidal cells (blue box). In Fet 2E, lack of Layer V large pyramidal neurons was observed as highlighted in the red boxed area compared to the control. Similarly, in Fet 3E frontal cortex, less small pyramidal neuronal population was observed in Layer III of the cortical plate. In layer V, although large pigmented cells are visible, they lack the characteristic apical dendrites of the large pyramidal neurons found in this layer as seen in the control brain (inset). Similar to Fet 1E, Fet 1M had clusters of small pyramidal neurons in Layer IV instead of Layer III as highlighted in the blue boxes. Sparse small pyramidal neurons in Layer III (yellow box) and large pyramidal neurons in Layer V (red box) were observed on Fet 2M frontal cortex compared to control. In Fet 3M frontal cortex, Layer III lacked the normal small pyramidal neuronal population. The cells highlighted in the yellow boxes might represent other neuronal cells present in this layer mainly stellate or basket cells. Similarly, Layer V lacked the distinctive medium to large pyramidal cells as seen in the control brain. The cells highlighted in the red boxes distinctly lack the apical dendrites of the pyramidal cells.

**Fig 6:**
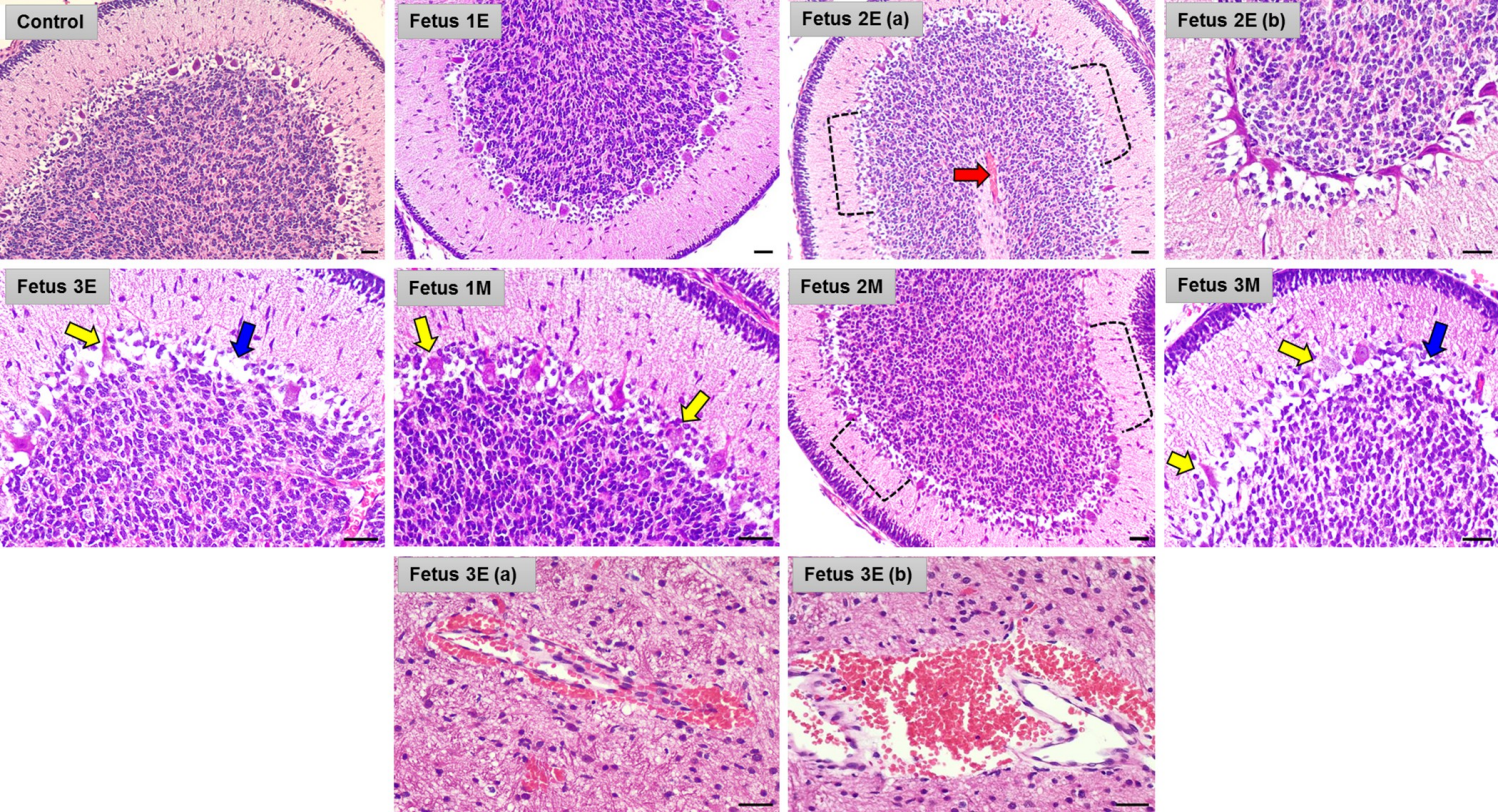
ZIKV inoculation results in structural cerebellar damage in early and mid-gestation infected fetal brain compared to the control. Representative images from H&E staining of control, early (**Fetus 1E, 2E, 3E**) and mid-gestation (**Fetus 1M, 2M, 3M**) cerebellum showing damaged and abnormal Purkinje cells in the ganglionic layer as marked by the yellow arrows whereas the blue arrows mark empty areas lacking Purkinje cells. The red arrow marks focal hemorrhage in the white matter of Fetus 2E cerebellum. In Fetus 2E (a) and Fetus 2M, the black dotted brackets highlight large areas in the ganglionic layer of the cerebellum lacking Purkinje cells. Fetus 3E (a and b) show large hemorrhagic areas in the cerebellar white matter. (Scale bar = 100μm).

H&E staining of the cerebellum showed a marked disruption of structural integrity of cerebellum of ZIKV fetuses compared to the control (**Fig 6**). In the early-gestation cohort, Fetuses 2E and 3E showed the most cerebellar defects including loss of Purkinje cells in the ganglionic layer of the cerebellum and focal hemorrhage in the granular layer compared to the control cerebellum. The cerebellum of Fet 1E had the fewest observable deficits. The cerebellum of Fet 3E had the greatest Purkinje cell layer damage coupled with hemorrhage in the white matter of the cerebellum.

In the mid-gestation cohort, the cerebellum of Fet 1M, 2M and 3M also exhibited loss of Purkinje cells. Fetus 3M showed the most damage to the cerebellar structure with marked loss of Purkinje cells in the ganglionic layer seen as dead or dying Purkinje cells and areas with no visible Purkinje cells in the ganglionic layer of the cerebellum.

### Gliosis

#### Microglia

**Fig 7:**
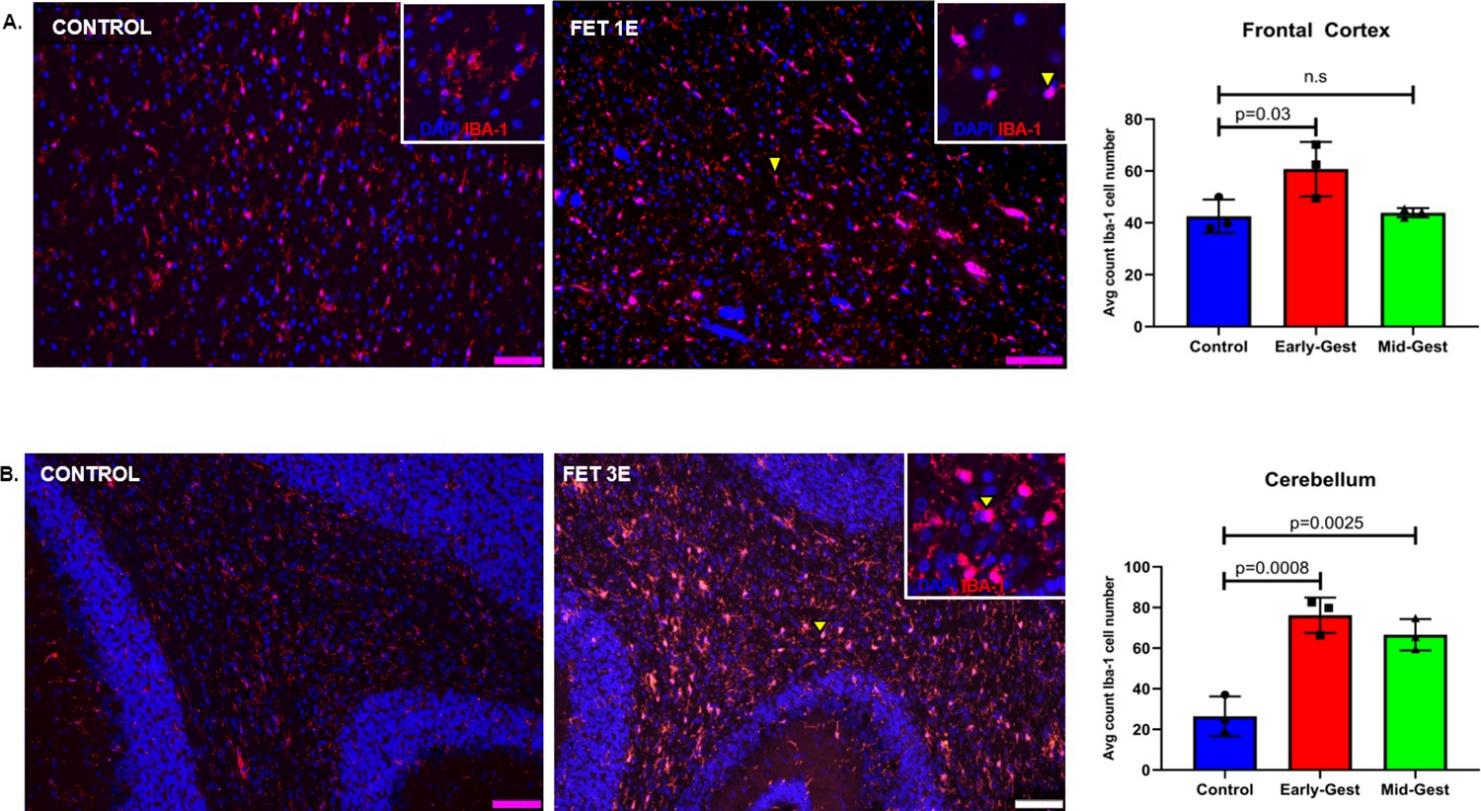
Immunofluorescence (IF) for neuroinflammatory marker for microglia (Iba-1) in the frontal cortex (**A**), and cerebellum (**B**) of control and ZIKV fetuses. In the white matter of the frontal cortex (**A**), significant increase in Iba-1+ reactive microglial cell (arrow and inset) population was observed in the early but not in fetal brains from mid-gestation ZIKV inoculated dams compared to controls. Microglial cells undergo morphological changes from resting to reactive state. Microglia in brains from control fetuses mostly appear in resting state (arrow, inset) where Iba-1 staining is seen concentrated in the neuronal processes which are more ramified compared to reactive or activated microglia in the brains from fetuses from ZIKV inoculated dams (arrow, inset) which are unramified with a larger cell body in an amoeboid shape where Iba-1 staining is most concentrated. Fetal cerebellum from both early and mid-gestation inoculated dams (**B**) had significantly more Iba-1+ reactive microglia (arrow and inset) in the granular layer compared to control brains (Scale bar = 50μm; Error bars depict SD, Dunnett’s multiple comparison test was done for statistical analysis, p<0.05 considered significant, n.s, non-significant).

In the frontal cortex, microglial cells (Iba-1) were counted in the white matter of each section. Early-gest infected fetal frontal cortices exhibited a significant increase in Iba-1+ cells in the white matter compared to the control brains (p = 0.03). Mid-gest fetal frontal cortices did not exhibit increased Iba-1 immunoreactive microglial population compared to the control fetuses (**Fig 7A**). In the hippocampus, Iba-1+ microglial cells were counted in the dentate gyrus (DG).

In the cerebellum, a significant increase in immunoreactive microglial cells was observed suggesting marked cerebellar inflammation in both the early and mid-gestation fetuses compared to the control (**Fig 7B**). The early-gestation ZIKV exposed fetal cerebellum exhibited 4-fold increase in Iba-1+ microglial cells (p = 0.0008) and the mid-gestation ZIKV exposed fetuses exhibited 3-fold increase in Iba-1 immunoreactive microglial cell population in the cerebellum compared to the control cerebellum (p = 0.0025).

#### Astrocytes

**Fig 8:**
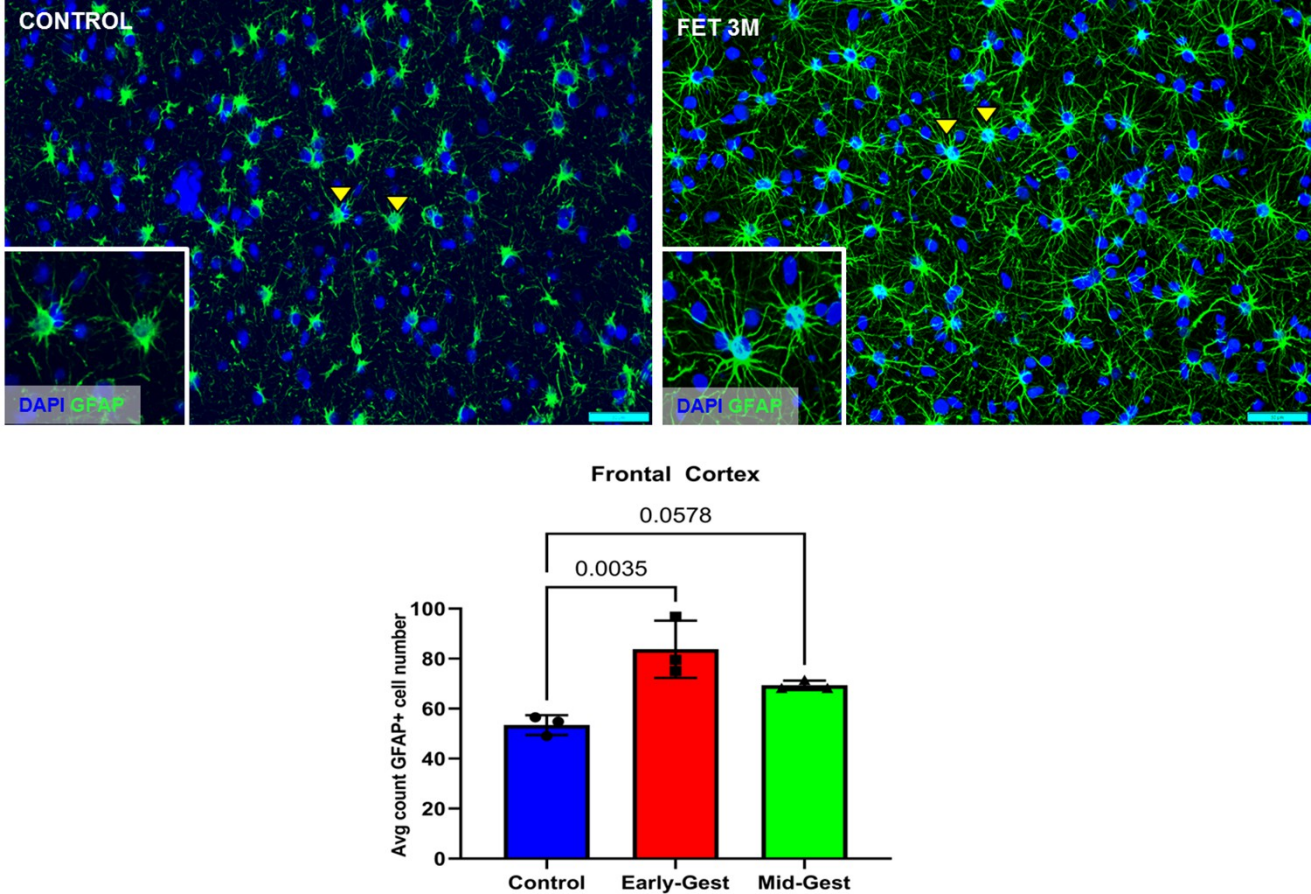
Immunofluorescence (IF) for GFAP representing astrogliosis in the frontal cortex of fetal baboons from early and mid-gestation ZIKV inoculated dams compared to the control fetal brains. In fetal frontal cortices from both early and mid-gestation ZIKV inoculated dams, significant increase in GFAP+ cell population was seen. GFAP immunoreactive cells also visually appeared to have increased GFAP staining intensity and morphological changes such as longer and more ramified cellular processes compared to control brains (arrow and inset). (Scale bar = 50μm; Error bars depict SD, Dunnett’s multiple comparison test was done for statistical analysis, p<0.05 considered significant).

In the frontal cortex, there was a significant increase in GFAP+ cells in both early and mid-gestation infected fetal brains compared to control (**Fig 8**). Significant increase in the astroglial cell population in the fetal brain at term is a mark of astrogliosis or reactive gliosis. Among the gestation groups, the early-gestation inoculation fetal cortex had significantly more GFAP positive cells compared to the control (p = 0.0035) than the mid-gestation group (p = 0.058). We also observed not only astrocytic hyperplasia but also hypertrophy, increased GFAP marker intensity and higher number of cytoplasmic processes suggesting changes in the astrocytic morphology and expression of cytoskeleton proteins which are the hallmarks of reactive gliosis [54]. There were no apparent changes in the hippocampal or cerebellar astrocytic populations in either early or mid-gestation infected fetuses despite the noted histological changes observed above.

### Olig-2 (Oligodendrocyte precursor):

**Fig 9:**
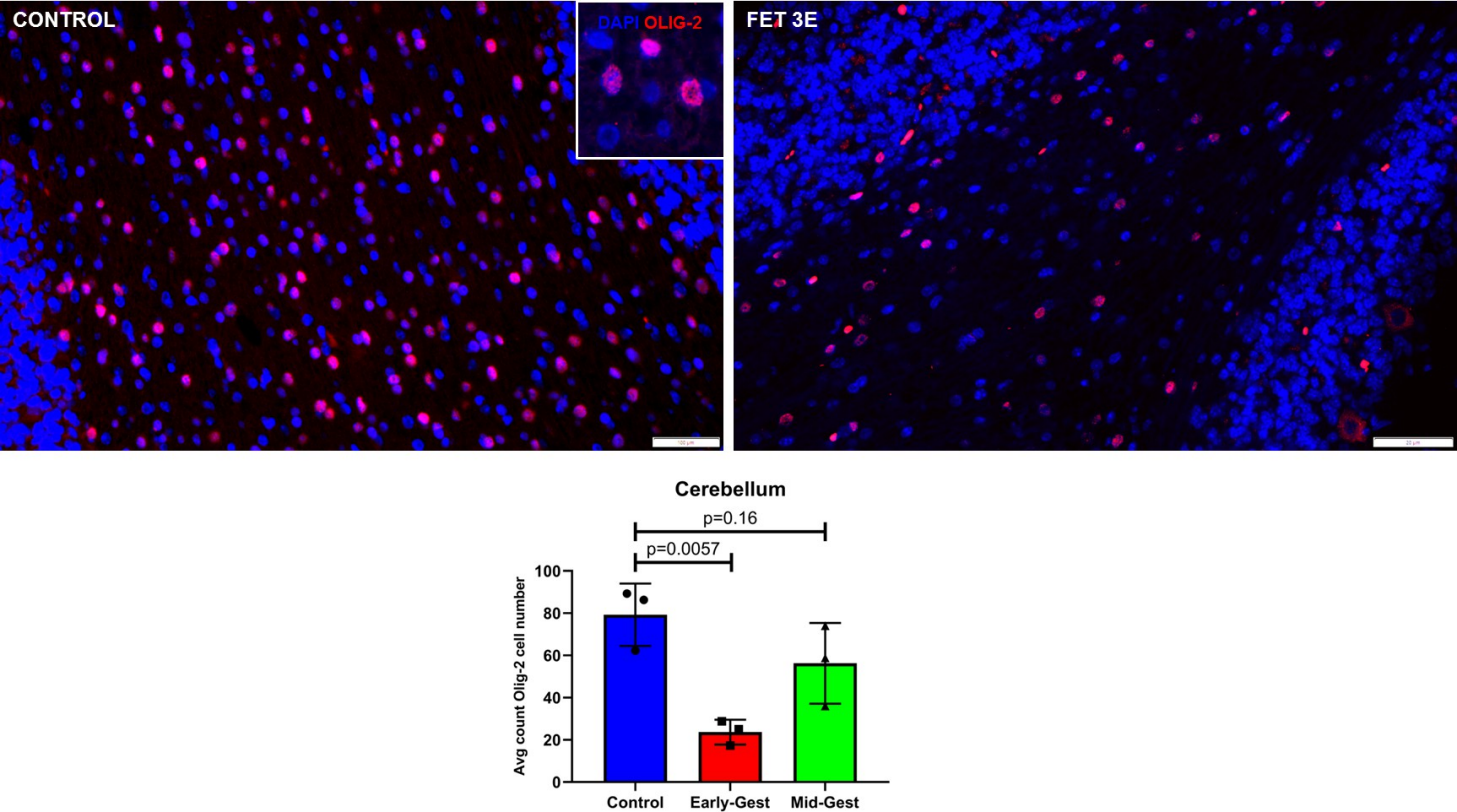
Immunofluorescence (IF) for Olig-2 (oligodendrocyte precursor) in the granular layer of the cerebellum. In the control cerebellum, numerous Olig-2+ cells (arrow, inset) could be seen in the granular layer whereas in fetal cerebellum from early-gestation ZIKV inoculated dams, significant decrease in Olig-2+ cell population were noted. However, fetal cerebellum from mid-gestation ZIKV inoculation dams did not show difference in Olig-2+ cell population compared to the control. (Scale bar = 100μm (control), 20μm (Fet 3E); Error bars depict SD, Dunnett’s multiple comparison test was done for statistical analysis, p<0.05 considered significant).

We evaluated the effects of ZIKV infection on the oligodendrocyte cell population in the cerebellum using Olig-2 immunostaining as a marker for oligodendrocyte precursor cell population which matures to become oligodendrocytes. Olig-2 immunostaining showed a significant decrease in the oligodendrocyte precursor cell population in the cerebellar white matter of ZIKV exposed fetuses compared to the control cerebellum (**Fig 9**). Early-gest cerebellum showed significant reduction in Olig-2 immunostained cells compared to the control (p = 0.005) whereas the mid-gest fetal cerebellar showed less but not significant reduction in Olig-2+ population (p = 0.16) compared to the control fetuses.

### Nestin and Doublecortin (DCX) in the hippocampus:

In order to determine the neurological impact of ZIKV infection on neural progenitor cells (NPCs) and newly differentiated and migrating immature neurons, we immunostained control and ZIKV exposed hippocampal sections using Nestin (NPCs) and Doublecortin (DCX, differentiation and migration) as markers. Hippocampal sub granular zone (SGZ) in the dentate gyrus (DG) is a neurogenic niche in all stages of brain development pre- and post-birth and throughout life. Hence, active neurogenesis takes place in the DG of the hippocampus with generation of NPCs as well as differentiation, migration and survival of the newly formed neurons.

**Fig 10:**
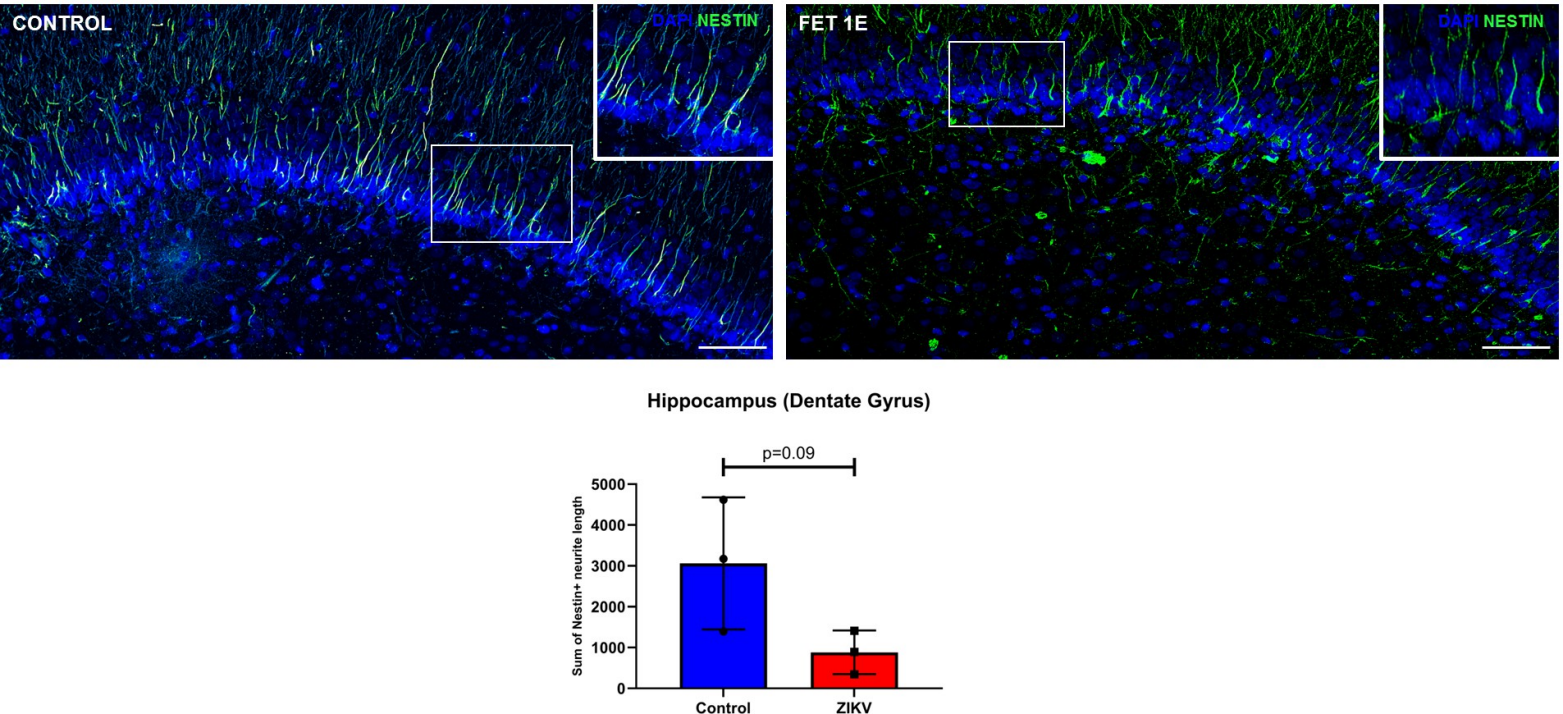
Immunofluorescence (IF) for neural progenitor cell (NPC) marker Nestin in the sub granular zone (SGZ) of the hippocampal dentate gyrus (DG). Control hippocampus show uniform and organized Nestin positive neurite processes of progenitor cells in the DG (box and inset). Hippocampal neurite lengths in the Nestin+ cells were reduced in fetuses from ZIKV inoculated dams with the appearance of non-uniform and disorganized distribution of Nestin+ neuronal processes of progenitor cells along the DG as compared to control hippocampi (inset) (Scale bar = 100μm; Error bars depict SD, unpaired *t*-test, p<0.05 considered significant).

Neurite length of Nestin positive cells in the DG were reduced in the ZIKV exposed fetuses compared to the control hippocampus (p = 0.09, **Fig 10**). The general appearance of neurites of the Nestin immunoreactive NPCs in the DG looked disorganized with loss of neurites, sparse and uneven distribution of Nestin^+^ cells with large gaps along the DG compared to the orderly distribution of symmetrical and long tracks of neurites of Nestin immunoreactive cells in control DG (**Fig 10**).

**Fig 11:**
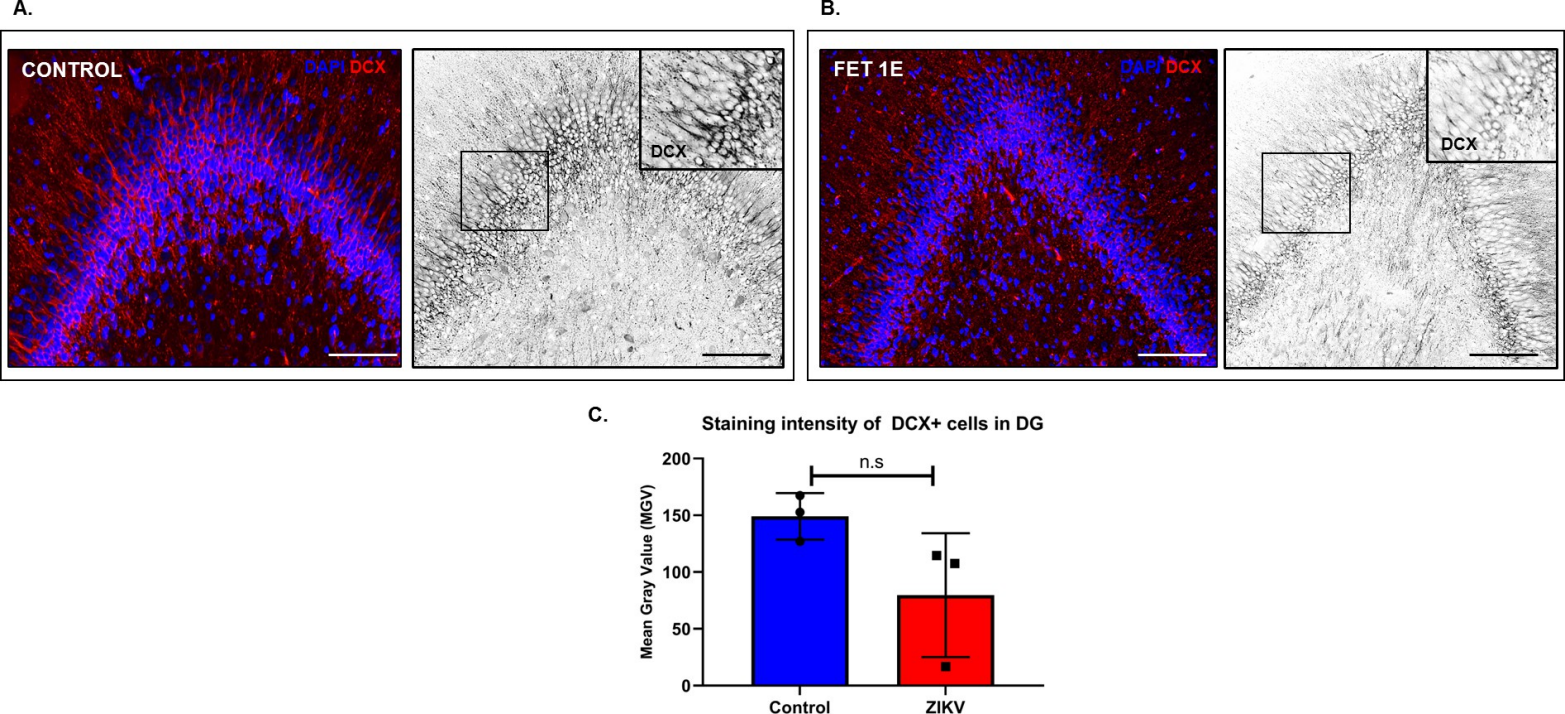
Immunofluorescence (IF) for neuronal migration marker doublecortin (DCX) in the dentate gyrus (DG) (**A**) and hippocampus (**B)** in fetal brains from ZIKV inoculated and control dams. Staining intensity of DCX+ neurons localized in the DG was measured as mean grey value (MGV). Control DG showed uniform distribution of DCX+ neurons with strong DCX staining in neuronal processes that appeared uniform and abundant (inset) whereas in fetuses from ZIKV inoculated dams, the DG had DCX+ cells with decreased DCX staining suggesting reduction in dendritic arborization of immature neurons along the DG (inset). (**C**) Difference in MGV of DCX+ neurons between ZIKV and control hippocampus was not statistically significant. (Scale bar = 100μm, Error bars depict SD, unpaired *t-*test, p<0.05 considered significant).

DCX immunostaining of ZIKV and control hippocampi showed a trend towards reduction in DCX+ staining intensity in the DG of ZIKV exposed fetuses compared to the control (**Fig 11**). DCX+ cells in the DG in the control hippocampi showed robust expression of the marker in the neurite processes (**Fig 11A**) whereas in the ZIKV exposed fetal hippocampus (**Fig 11B**), staining intensity decreased in the DCX immunoreactive cells suggesting possible reduction in dendritic arborization due to ZIKV exposure. DCX+ arborization in ZIKV hippocampus also appeared non-uniform with gaps along the DG with asymmetrical and mis-patterned neuronal projections compared to the control (**Fig 11A and 11B insets**).

### Placental and uterine histology and IF

**Fig 12:**
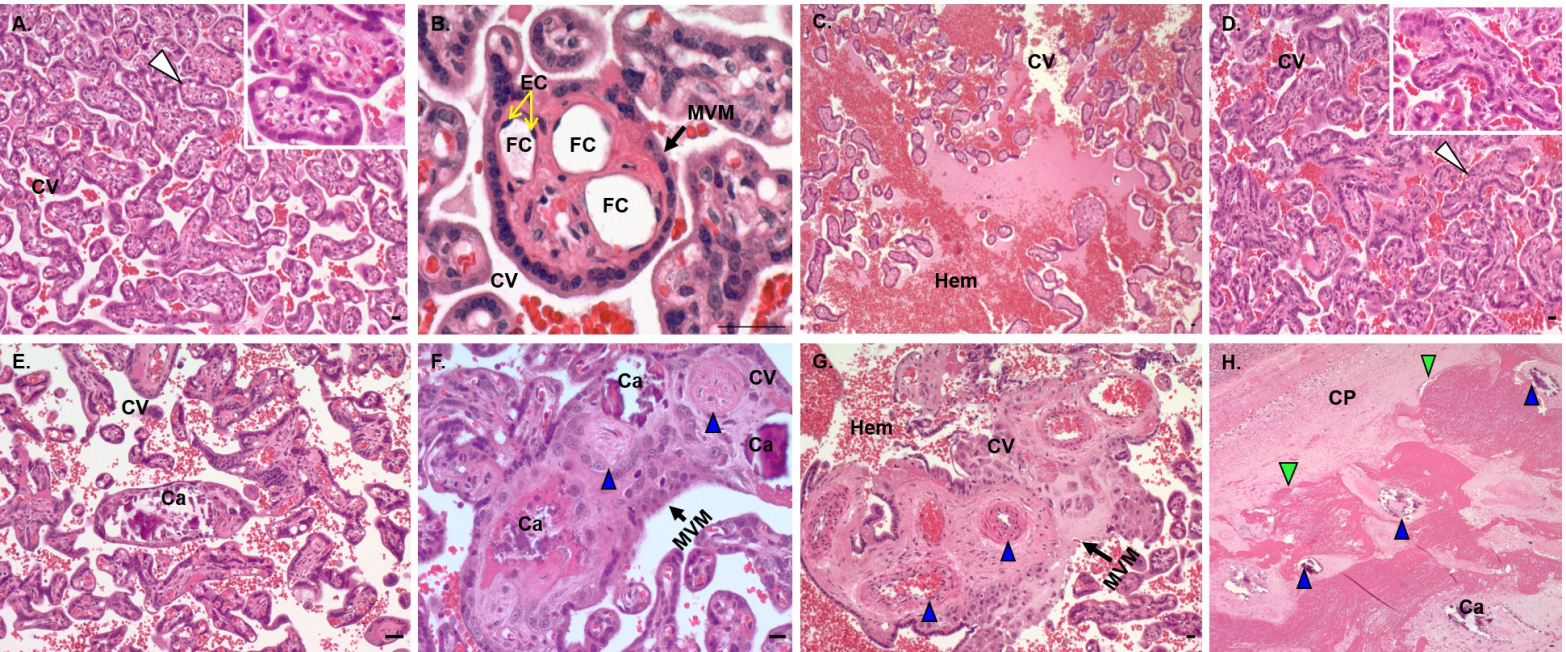
Placental histopathology in control dams and dams inoculated with ZIKV. (**A, B**) Control placenta with normal chorionic villi structure and mature vascular villi surrounded by intact MVM and containing numerous fetal capillaries (arrowhead, inset) and fetal capillaries lined with endothelial cells (**B**). (**C, H**) Placenta from ZIKV inoculated dams showed multiple changes in the villi and vascular structure such as immature and avascular villi (**D**; arrowhead, inset), hemorrhage (**C, G**), fetal capillary thickening, thrombosis and mineralization (**F, G, H**; blue arrowhead), disruption of MVM (**F, G**) and fibrin deposits (green arrowhead) surrounding fetal vessels undergoing thrombosis and mineralization (**E, F, H**). **Fig C, H** are from Dam 1E placenta and **Fig D, E, F and G** are from Dam 3E placenta. CV, chorionic villi; EC, endothelial cells; FC, fetal capillaries; Hem, hemorrhage; MVM, microvillous plasma membrane; Ca, calcification; CP, chorionic plate. (Scale bar = 50μm).

The histopathology of ZIKV infection in our early and mid-gestation inoculated groups showed pathology indices compared to the control (**Fig 12**). In the control placenta, the chorionic villi were seen as mature late gestation villi surrounded by intact microvillous plasma membrane (MVM) which is the outermost polarized plasma membrane of the syncytiotrophoblast directed toward maternal blood in the intervillous space. These control villi had multiple unobstructed fetal capillaries lined with endothelial cells and few fibrin and calcium deposits throughout the villi on the fetal side (**Fig 12A** and **12B**). In ZIKV groups, noted pathologies observed included occasional observation of immature villi with reduced branching and less terminal villi intermixed with intermediate villi that were not noted in control placenta possibly indicating delayed villous maturation (**Fig 12D**). Avascular villi (villi without fetal capillaries) (**Fig 12D inset**), disrupted MVM **(Fig 12F and 12G)**, partially or fully thrombosed vessels with calcium mineralization and fibrin deposits (**Fig 12E, 12F and 12G**) and thrombosed fetal blood vessels in the chorionic plate undergoing mineralization and surrounded by fibrin deposits (**Fig 12H**) were also observed in placentas from ZIKV inoculated dams that were not observed in control placenta. Dam 1E had notable hemorrhage in the intervillous space (**Fig 12C**). However, considering that term primate placentas have similar localized pathologies, we cannot rule out that our observations were due to sampling site of each placenta.

**Fig 13:**
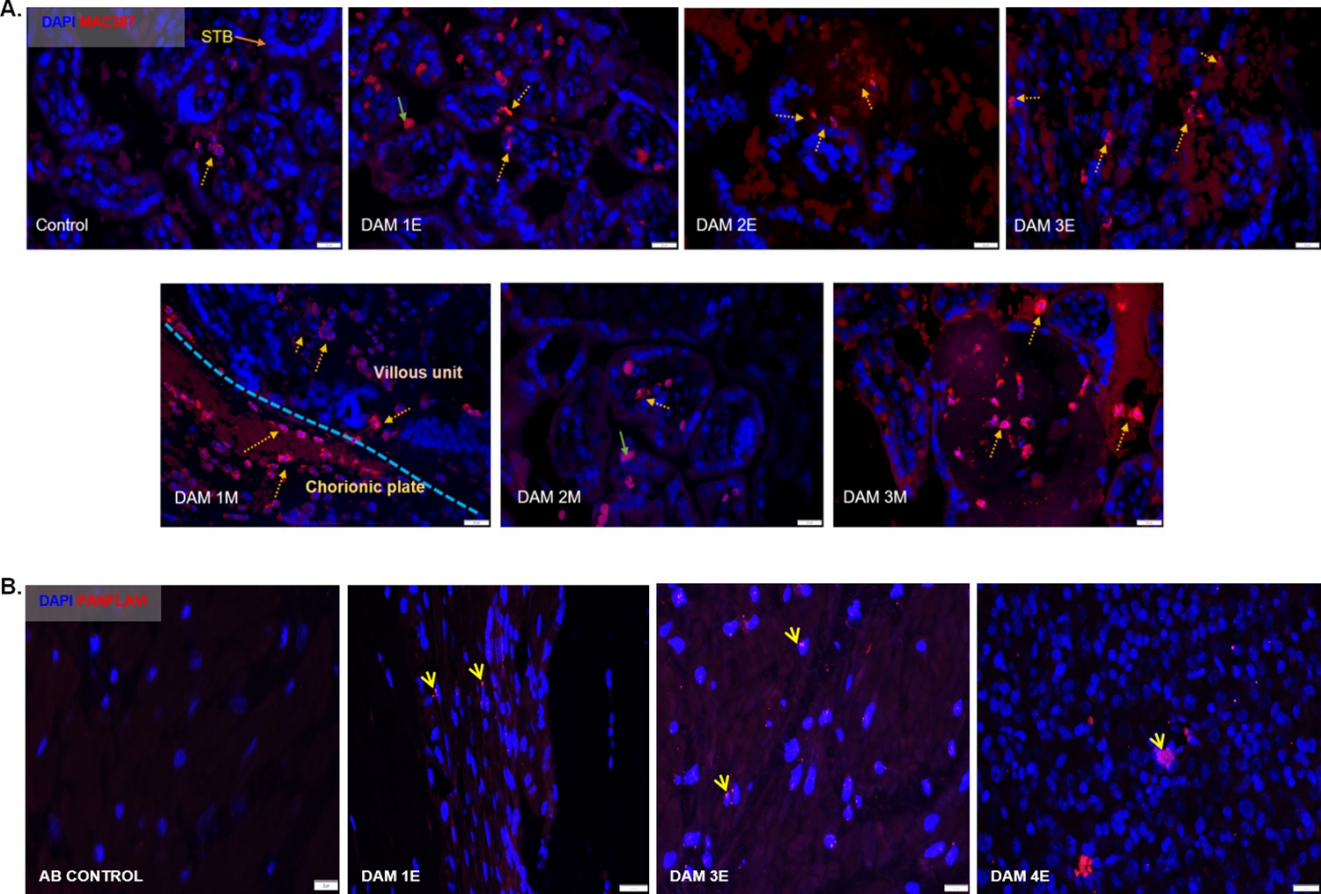
Immunofluorescence (IF) for placental macrophages (MAC387) and ZIKV (pan flavivirus) in the uterus. (**A**) Macrophage staining in the placenta indicated with dotted yellow arrows was seen mostly in the villous and intervillous space in the placenta in Dams 1E, 2E, 3E, 2M and 3M and in chorionic plate on the fetal side in dam 1M (blue dotted line separating the fetal and villous sides). Only occasional macrophages were observed in Dam 1E placenta and abundant macrophages were observed in Dam 3M within villi compared to the control placenta (green arrows denote auto-fluorescing red blood cells). (**B**) Pan-flavivirus immunofluorescence staining in the uterus. ZIKV IF was observed in the endometrial stromal cells in Dams 1E, 3E and 4E (yellow arrows). Dam 4E had widespread ZIKV IF staining with clusters of ZIKV positive cells. None of the mid-gestation inoculated dams showed ZIKV IF staining in the uterus. (Scale bar = 20μm).

Placental sections from early and mid-gestation inoculated dams exhibited notable MAC387 (macrophage) positive cell populations in the villous, intervillous space (IVS) on the fetal and maternal side compared to control placenta which exhibited few MAC387+ cells (**Fig 13A**). One distinction between MAC387+ cells in the early vs mid-gestation inoculated placentas was the location of these cells in the two groups. In the early-gestation inoculated placenta (Dam 1E, 2E, 3E), the MAC387+ cells were found interspersed in the terminal villi and IVS whereas in the mid-gestation inoculated placenta (Dam 1M, 2M and 3M) the MAC387+ cells were concentrated in the chorionic plate than villi on the fetal side to a greater degree (**Fig 13A**).

Three out of four early-gestation inoculated dams (Dams 1E, 3E and 4E) were positive for ZIKV IF in the uterus (**Fig 13B**). Uterus of these dams also tested positive for ZIKV RNA by q-PCR (**Table 3**). ZIKV IF was detected primarily in the endometrial stromal cells. In the case of Dam 4E, ZIKV IF was seen widespread and more diffused in the endometrial stromal cells. Dam 4E also aborted 5 days post inoculation. Dams in the mid-gestation cohort were negative for ZIKV IF in the uterus. ZIKV IF was not detected in the placenta samples collected from the dams at the time of necropsy.

## Discussion

The 2015 ZIKV epidemic in Brazil revealed devastating clinical consequences to the fetus resulting from ZIKV infection during pregnancy. CZS includes a wide array of neurological and developmental disorders in the fetus and the infant post-birth. Therefore, studying ZIKV and its pathophysiology remains crucial in understanding how viruses like ZIKV cross the placental barrier to infect the developing fetus, causing permanent damage to the developing brain. In the present study we examined the long-term pathological outcome in term gestation fetuses resulting from ZIKV infection during early and mid-gestation, since a more severe CZS phenotype is observed clinically in human fetuses exposed to ZIKV in mothers infected earlier in gestation [4]. Our findings using the Olive baboon as a NHP model show pronounced CNS pathologies in fetuses from ZIKV exposed dams infected early and mid-gestation consistent with reports in human fetuses.

Following ZIKV inoculation, all early-gestation (n = 4) and two of three mid-gestation dams exhibited RNAemia within the first week post-inoculation (4–7 dpi). RNAemia at any other time points up to the day of necropsy was not observed in any of the pregnant dams. This differs from the reports in pregnant macaques where prolonged or re-emergent viremia for several weeks up to 70 dpi has been widely reported [24,25,28–30,41,43,55]. While prolonged viremia (46 to 53 days) has been reported in pregnant women [56–59], this appears to be uncommon. One study (PMID: 31449521) reported prolonged viremia in two pregnancies (out of 26 cases studied), two studies of prolonged viremia (PMIDs: 27028667; 27959695) were case reports of single patients and one study (PMID: 27479770) found five cases out of the entire USA 2016 ZIKV Pregnancy Registry of 1,297 ZIKV pregnancies. Further, prolonged viremia during pregnancy in women does not associate with severity of CZS.

We did not detect ZIKV vRNA in near term placental samples, cord blood, amniotic fluid or fetal tissues collected during. While we did find high titers of ZIKV IgG in cord blood, we did not detect ZIKV IgM. The presence of high and likely neutralizing titers of ZIKV IgG in the fetus is consistent with the well-known transplacental transfer of maternal IgG via the FcγR and fetal passive immunization. In our previous study, we observed vertical transfer (placenta, fetal tissues) in the Olive baboon between 7 and 21dpi, therefore, it is possible that vertical transfer of ZIKV in the present cohort occurred early post-inoculation and resolved by term, possibly due to neutralization of ZIKV IgG in the fetal compartment or from ZIKV-induced death of infected fetal cells without efficient viral replication. However, we also cannot rule out the possibility of ZIKV in tissues other than the ones we collected at necropsy. Notably, vRNA persisted in the various lymph nodes in one early gestation and three mid-gestation dams. While two of the early-gest infected had persistent and notable ZIKV viral immunostaining in uterine tissue samples collected at term gestation, none of mid-gestation dams exhibited uterine ZIKV RNA. Thus, at the present time we do not know if infectious ZIKV virions remained in the uterus at term. However, presence of ZIKV in uterus from early-gestation infection with PR strain has also been reported in rhesus macaques [42].

ZIKV infection during pregnancy results a variety of fetal or infant neuropathology described in humans and several animal models including NHPs, with the most notable difference to date being the observation of microcephaly in humans that had not been replicated in NHPs [25,27,28,31,32]. In the present study in the Olive baboon, there was a notable difference in gross brain structure in near term fetuses from ZIKV exposed dams compared to controls where structural differences in gyral and sulcal patterns were observed. Baboon cerebral development is similar to humans and baboons have the larger brain size with the average cerebral volume at least twice as large as that of the most commonly used NHP, the rhesus macaque. Baboon brains also exhibit the highest cerebral gyrification index (GI) with all primary human cortical structure homologues [60–62]. One of the most important stages of brain development in primates is the orderly process of gyrogenesis where a smooth, lissencephalic fetal brain develops a characteristic pattern of gyri and sulcal folding [63].

The abnormal features of gyri/sulci formation in the fetal brains from ZIKV inoculated dams included asymmetric gyrogenesis and sulcal formation, enlarged gyri, abnormal sulcal length and depth, absence of sulci, widening of the sulci specifically lateral and superior temporal and inter-hemispheric fissures (Fetus 3E and 3M) giving these brains a misshapen and flaccid appearance. Abnormalities of the gyral patterns resulting in polymicrogyria, lissencephaly or pachygyria is a hallmark of ZIKV infected cases of human microcephaly [64,65]. We also observed increase in occipital lobe surface area compared to the frontal lobe in fetuses from ZIKV exposed dams. In humans, ZIKV affects both frontal and occipital cortical development resulting in cognitive and visual defects even in cases lacking overt microcephaly.

Radial glial fibers aid in gyri/sulci formation [66] [67]. In our previous study in baboons infected with ZIKV at mid-gestation with evidence of vertical transmission, we reported loss of RG fibers in the frontal cortex of the fetus at 21 dpi [21]. As such, loss of RG fibers would be predicted to impair brain gyri/sulci formation and structure. In our prior study, we hypothesized that RG loss, including RG fibers, would have resulted in a less folded brain. Our finding that not all gyri and sulci exhibited abnormalities supports that ZIKV infection in the CNS is likely restricted or focal (similar to that described for placental cotyledons), thus only gyri and sulci targeted by ZIKV would seemingly exhibit defects in development. The size and extent of mammalian cortical folding affects cognition and sensorimotor skills and disruptions in cortical growth and folding leads to neurological disorders such as autism and schizophrenia [67–69]. The fetal cortex regions where we observed most disturbances in fetuses from ZIKV exposed dams (central sulcus; supramarginal, superior temporal, middle temporal, angular and occipital gyri) are important for motor, sensory, visual and auditory functions [70]. Therefore, the potential for the development of long-term neurocognitive deficits in infants born with disrupted cortical growth and folding in the absence of microcephaly remains high.

In macaques, although significant neuropathology has been described in response to maternal [28,31,32] or intra-amniotic [27] ZIKV infection, minor or no abnormalities in cortical folding has been noted. There was a description of one fetus missing an occipital gyrus in one hemisphere [28]. Another study using postmortem MRI analysis observed no differences in cortical folding or structural abnormalities [41], although noting microcephaly (reduced brain volume) in one infant from a ZIKV infected dam. Other macaque studies have published smaller brain volume as well as head circumference 2–3 SD below the mean without evidence for gross brain structure anomalies [25,28].

Histopathology using H&E staining revealed disturbances of the six-layered cortical plate (CP) in the frontal cortex in a fetus from ZIKV exposed dams. These disturbances observed were mostly in Layer III and V of the cortical plate which consists of mainly small and large pyramidal cells respectively which are the main output neurons of the cerebral cortex. [63]. As noted above, in our prior study in baboons infected with ZIKV at mid-gestation, we reported decreased cortical NPCs as well as cortical plate neuronal disorganization at 21 dpi [21]. Radial glial fibers, in addition to aiding in brain folding, provide the necessary scaffolding for neuronal and NPC migration to the cortical plate. Thus, our prior observation of RG and RG fiber loss would predict noted disruption in the cortical plate in the present study. While we did not note changes in NPC (Nestin+) or immature neurons (DCX+) in the cortex in the present study, differences in the distribution and appearance of NPCs and immature neurons along the dentate gyrus (DG) in the hippocampus as well as a trend towards reduction in neurite length of Nestin+ cells and of dendritic arborization of DCX+ cells on the DG. We also observed non-uniform and asymmetrical arrangement of both Nestin and DCX staining with gaps along the DG compared to the control DG. Such changes in dendritic arborization and appearance has been reported in a ZIKV pigtail macaque study [32].

We observed a significant decrease in oligodendrocyte precursor cell population in the early-gestation cerebellar white matter. In humans and macaques, ZIKV infection is known to cause white matter damage and reduction in myelination [32,71,72] which could result from the observed loss of oligodendrocyte precursor population in cortical white matter.

Similar to the frontal cortex, the cerebellar histological staining revealed considerably more damage to the cerebellum of fetuses from ZIKV exposed dams, mainly a significant loss of Purkinje cells and focal hemorrhage in the white matter of the cerebellum. Besides its role in motor circuits, recently several studies have focused on the non-motor circuit role of cerebellum mainly in cognitive functions such as language, behavior and emotions [73,74]. Cerebellum growth abnormalities such as absence, underdevelopment or atrophy are frequently seen in infants with CZS [11] and children born with ZIKV CZS are reported to display hyperactivity, severe irritability and self-injurious behavior which are also characteristics of behavioral disorder due to cerebellar damage [75,76].

In addition to the observed structural damage in fetuses from ZIKV exposed dams, a significant increase in Iba-1 staining, a marker for microglia, was observed in the frontal cortex and cerebellum. Reactive microglia, arising in situations of inflammation and injury, exhibit a change in morphology, transitioning from a highly ramified structure to one with a denser soma, shorter thick processes and higher expression of Iba-1 [77]. Reactive microglia were mostly observed in white matter of the frontal cortex and cerebellum with significantly higher numbers of Iba-1+ cells in the white matter of fetuses from both early and mid-gestation ZIKV exposed dams. Fetuses from early gestation ZIKV exposed dams exhibited significantly more Iba-1+ cells in the frontal cortex compared to fetuses from mid-gestation infected dams, consistent with a greater degree of neuropathology from early gestation ZIKV inoculation. Similar to increased presence of reactive microglia in the frontal cortex, astrogliosis was observed in the cortex in fetuses from dams infected with ZIKV, greater gliosis observed in fetuses from dams infected at early gestation compared to mid-gestation. Astrogliosis has been observed in human microcephalic cases and in NHP animal models in response to ZIKV infection [28,32,78]. Reactive astrocytes undergo morphological changes in their active state by increasing in size and with more cytoplasmic processes due to increase in intermediate filaments, particularly GFAP, which is detected as an increase in GFAP staining intensity [79]. Collectively, the noted increase in reactive microglia and astrocytes suggests neuroinflammation may contribute to the overall neuropathological outcome, in particular from early gestation infections. Our prior study also reported significant increase in microglia immunostaining and astrogliosis in the initial 21dpi [21]. As such, neuroinflammation is likely an early event post-ZIKV infection and may contribute to the overall neuropathology.

The placenta plays a critical central role in fetal development and outcome. One of the questions still unanswered is the role placental pathology such as vascular insufficiency and inflammation due to ZIKV infection. Studies in humans and pregnant macaques have shown a range of placental pathology resulting from ZIKV infection ranging from no discernable pathology to mild through severe pathology including deciduitis, chorioamnionitis, villitis and calcifications. Studies by Hirsch et al [26] and Martinot et al [28], found extensive placental damage in their macaque models including multiple placental infarctions, chorioamnionitis and villitis. They also showed vascular pathologies including vasculitis, thrombosis and vascular collapse resulting in disturbances in transplacental oxygen transfer and decreased fetal oxygenation. Fetal hypoxia can impact fetal CNS development and may alter fetal CNS susceptibility to ZIKV [36,41,43]. In the present study the most notable observation in placentas of both early and mid-gestation inoculated groups was macrophage infiltration in both the maternal and fetal placental compartments that was not observed in control placentas. The placentas from ZIKV exposed pregnancies also exhibited observations of immature villi, disruption of the microvillous plasma membrane of the syncytiotrophoblast layer and fewer fetal villi capillaries, capillary thickening and thrombosis and calcium and fibrin deposits in villi and in fetal vessels. By late gestation, there is a single syncytiotrophoblast layer separating maternal and fetal compartments and any loss of integrity of this layer provides a mechanism for vertical transfer of either the virus itself or of ZIKV infected maternal immune cells. Similar histopathological changes have been reported in placentas of ZIKV infected women as well [80,81]. However, one caveat regarding the changes in placental histopathology, our data may not reflect the entire placenta due to sampling restriction for histology. Placentas from ZIKV infected dams may therefore exhibit a mild to moderate pathology or simply reflect the site chosen for histology since these pathologies have also been described in term placentas from uncomplicated human and macaque pregnancies.

In conclusion, this study in pregnant baboon with ZIKV inoculation at early and mid-gestation recapitulates many of the brain pathologies associated with human cases. While vertical transfers in these studies of long gestation NHPs are hard to confirm, it is also the case in most human pregnancies, where most often ZIKV induced neurodevelopmental abnormalities are not obvious until birth if the fetus remains viable. Remarkably, all the fetuses sustained some CNS injury with ZIKV exposure *in utero* with those in the early gestation inoculation group sustaining the most on a cellular level as reported in human ZIKV cases where early-gestation ZIKV infection was linked with the most severe CZS outcome compared to late gestation infection. We now know from human cases that subtle fetal injuries *in utero* in the absence of microcephaly and overt CZS can still have detrimental long term cognitive, behavioral, emotional and social consequences. This study highlights the importance of studying direct or indirect inflammation in the fetal CNS, in the development of neuropathology and the mechanisms via which ZIKV induces long-term neurodevelopmental deficits. We acknowledge the limitation of the study in its small sample size (n = 4 early-gest, n = 3 mid-gest) which could limit our capacity to model a disease with highly variable pregnancy outcomes. However, other NHP studies have used smaller cohort sizes for their studies [25,26,29,32,41–43] for presumably economical and ethical reasons similar to ours. Indeed, even in limited capacity, our model replicated brain pathology seen in ZIKV-infected human pregnancies and therefore, provides a valuable model to study this disease mechanistically and for therapeutic interventions. Future studies need to address cognitive and behavioral outcomes and post-birth CNS development and maturation to fully assess the impact of ZIKV infection during pregnancy on the offspring and to develop interventions to lessen the impact of ZIKV infection on the childhood development.

## Materials and methods

### Ethical statement

All experiments utilizing baboons were performed in compliance with guidelines established by the Animal Welfare Act for housing and care of laboratory animals and conducted in accordance with and approval from the University of Oklahoma Health Sciences Center Institutional Animal Care and Use Committee (IACUC; protocol no. 101523-16-039-I). All studies with ZIKV infection were performed in Assessment and Accreditation of Laboratory Animal Care (AAALAC) International accredited ABSL2 containment facilities at the OUHSC. Baboons were fed standard monkey chow twice daily as well as receiving daily food supplements (fruits). Appropriate measures were utilized to reduce potential distress, pain and discomfort, including post-CSF collection analgesia. All animals received environmental enrichment. ZIKV inoculated animals were caged separately but within visual and auditory contact of other baboons to promote social behavior and alleviate stress. At the designated times post inoculation (**Table 1**), the animals were euthanized according to the recommendations of the American Veterinary Medical Association (2013 panel on Euthanasia).

### Animals

Adult timed pregnant female olive baboons (early (E) gestation n = 4: Dams 1-4E; mid (M) gestation n = 3: Dams 1-3M) were utilized for this study. Control dams (n = 3) were inoculated with saline and subjected to the same experimental regimen as the ZIKV inoculated dams. At the end of the study for each animal (167–169 dG [0.9 gestation] normal term ~183days), dams were sedated with ketamine, all maternal samples obtained as well as ultrasound measurements, then the animal rapidly euthanized with euthasol. A Cesarian (C) section was quickly performed, cord blood obtained and the fetus euthanized with euthasol. Maternal and fetal tissues were rapidly collected and samples were both fixed with 4% paraformaldehyde and frozen on dry ice (stored at—80°C) for each tissue.

An additional n = 10 control fetal brains (0.9G), obtained from dams following C-section under isoflurane anesthesia on another IACUC approved study, were used for assessing effect of ZIKV inoculation on gross brain structure. All females were multiparous with history of successful prior pregnancies. All dams used in this study were determined to be seronegative for West Nile Virus prior to infection and in response to ZIKV [49].

- **Virus stocks, inoculation and sample collection**- The PR isolate (PRVABC59) was obtained from ATCC (VR-1843) and was sequence authenticated. Viral stocks of the PR isolate were amplified in African Green Monkey Kidney cells (Vero cells, ATCC CCL-81) and quantitated by standard viral plaque assay. The viral stock (4x10^^^5 pfu/ml) was thawed and diluted 10-fold in PBS on the day of the challenge. Animals were anaesthetized with an intramuscular dose of Ketamine (10 mg/kg) before all procedures (viral inoculation, blood, salivary and vaginal swabs and urine collection). Timed pregnant female baboons were inoculated subcutaneously at the mid-scapular area with a single clinically relevant dose of 10^4^ plaque forming units (pfu; 1 ml volume per dose) of the Puerto Rican ZIKV isolate (PRVABC59). The dosage used to inoculate the animals in our study is based on the previous works done in mosquitoes carrying WNV and DENV, where it was estimated that mosquitoes carry 1x10^4^ to 1x10^6^ plaque forming units (PFU) of the virus [82], from a study evaluating Brazilian ZIKV in a bite from *Aedes aegypti* mosquito [83] and from a study of mosquito transmission of ZIKV in rhesus monkeys [84]. The pregnant females were inoculated near early-gestation (between 55–58 days of gestation [dG]) and mid-gestation (between 92–97 dG; term is approx. 181 dG; the overall approach is detailed in **Table 1**). Maternal blood samples, vaginal and salivary swabs were obtained on the day of inoculation (day 0) as well as ultrasound evaluation of fetal viability. Whole blood was collected into EDTA tubes. Saliva and vaginal samples were collected by cotton roll salivette. The sampling procedure for each dam is detailed in **Table 1**. Observed clinical signs post-inoculation with ZIKV for each dam is detailed in **Table 2**. For early-gestation group, samples (blood, vaginal and salivary swabs) were obtained at days 0, 4, 7, 14, 21, 42, 80 and 115 post inoculation and amniotic fluid and maternal-fetal tissue were collected at the termination of the study (169–172 dG). For mid-gestation group, samples (blood, vaginal and salivary swabs) were collected at days 0, 4, 7, 14, 21, 42, 73 post inoculation and amniotic fluid and maternal-fetal tissue were collected at the termination of the study (167–169 dG).
- Complete blood counts (CBCs): CBCs were evaluated for all females on EDTA-anticoagulated whole blood samples collected on day 0 and subsequent days-post inoculation as shown in the experimental timeline (Idexx ProCyte DX hematology analyzer; Idexx laboratories, ME). CBC’s included analysis for red blood cells (RBCs), hemoglobin, hematocrit and platelet count. RBC, hemoglobin and hematocrit numbers did not show any differences pre-and post ZIKV inoculation for any of the infected females. Platelet counts did not change in response to ZIKV inoculation in any dam.
- **One-Step quantitative reverse transcription PCR–**Primers and probes used for qRT-PCR were designed by Lanciotti et al [85] (**Table 5**). RNA was isolated from maternal and fetal tissues (**Table 3**) using QIAamp cador pathogen mini kit (Qiagen, Valencia, CA). ZIKV RNA was quantitated by one-step quantitative real time reverse transcription PCR using QuantiTect probe RT-PCR kit (Qiagen) on an iCycler instrument (BioRad). Primers and probes were used at a concentration of 0.4 μM and 0.2 μM respectively and cycling conditions used were 50°C for 30 min, 95°C for 15 min followed by 40 cycles of 94°C for 15 s and 60°C for 1 min. Concentration of the viral RNA (copies/milliliter for fluids and copies/mg for tissues) was determined by interpolation onto a standard curve of six 10-fold serial dilutions (10^6^ to 10^1^ copies/ml)) of a synthetic ZIKV RNA fragment available commercially from ATCC (ATCC VR-3252SD). The cutoff for limit of detection (LD) of ZIKV RNA was 1x10^2^. Each viral load reported is the average of 3 replicates.

**Table 5:** Primer/Probe sets for the detection of ZIKV by one step qRT-PCR.

	Primers	Genome Position	Sequence (5`-3`)
1	ZIKV 835 Forward	835–857	TTGGTCATGATACTGCTGATTGC
	ZIKV 911 Reverse	890–911	CCTTCCACAAAGTCCCTATTGC
	ZIKV 860-FAM Probe	860–886	CGGCATACAGCATCAGGTGCATAGGAG
2	ZIKV 1086 Forward	1086–1102	CCGCTGCCCAACACAAG
	ZIKV 1162 Reverse	1162–1139	CCACTAACGTTCTTTTGCAGACAT
	ZIKV 1107-FAM Probe	1107–1137	AGCCTACCTTGACAAGCAGTCAGACACTCAA

- **ZIKV ELISA—**ZIKA specific IgM and IgG antibody responses were assessed in the serum samples using the commercially available anti-ZIKV IgM (#ab213327, Abcam, Cambridge, MA) and IgG (#Sp856C, XpressBio, Fredrick, MD) ELISA kits. Briefly, a 1:100 for IgM and 1:50 for IgG serum dilution was performed in duplicate and added to the pre-coated plates available in the kits. The assays were performed using the manufacturer’s instructions and the absorbance was read at 450 nm for IgM and 405 nm for IgG antibodies in the serum.
- **Fetal CNS and placental Immunohistochemistry/Immunofluorescence–**Following removal, fetal cerebrum and cerebellum were divided mid-sagittal and frontal cortex and hippocampus dissected out of each cerebral hemisphere. one half of each tissue was rapidly frozen on dry ice and stored at -80°C and the other half fixed in 4% paraformaldehyde for 48 hours, transferred to 70% EtOH and paraffin embedded following paraffin processing and embedding protocols. The identical region of the frontal cortex was selected from each fetal brain and subjected to serial coronal sectioning (5 μm) along with the cerebellum and hippocampus. Fetuses (n = 3) delivered via C-section from late gestation pregnant baboons were used as controls for this study.

Histological staining in the form of H&E stain was done on fetal frontal cortex, cerebellum, hippocampal and placental tissue sections from all ZIKV and control fetuses. Sections were visualized using a brightfield microscope (Olympus B40x) equipped with a SPOT 5 MP digital camera with SPOT 5.3 imaging software (Sterling Heights, MI).

For immunocytochemical and immunofluorescent labelling, sections every 150 microns were selected for immunolabeling for a total of 4 sections per fetus. For dual Immunofluorescence labeling (IF) on paraffin sections, after deparaffinizing and rehydrating protocol, HIER (heat-induced epitope retrieval) was performed in a Retriever 2100 with citrate-based antigen unmasking solution (#H-3300, Vector Laboratories, Burlingame, CA). After retrieval, slides were rinsed in 1XPBS and treated with 1% NaBH4 solution to reduce auto-fluorescence then blocked for two hours in blocking solution at room temperature in the humidity chamber followed by overnight incubation in primary antibodies (Iba-1 Rabbit polyclonal 1:100, NBP2-16908; Nestin; mouse monoclonal 1:100, NBP1-92717; GFAP, Rabbit polyclonal 1:500, #12389S; DCX, Rabbit polyclonal 1:200, #4604S, Cell Signaling, Danvers, MA; Olig-2, Rabbit polyclonal 1:500, AB9610, Millipore, CA). Sections were incubated for an hour the next day at room temperature in dark in goat anti-mouse Alexa 568, goat anti-rabbit Alexa 568 and goat anti-mouse Alexa 488 1:200 (Life Technologies, Carlsbad, CA) based on the combinations of primary antibodies used for the immunostaining. After PBS washes, sections were incubated for 30 min in dark at room temperature in Prolong Gold with DAPI (Life technologies, Carlsbad, CA), cover slipped and cured for 24 h before visualizing using fluorescent microscope (Olympus BX43). Control reactions were performed where the primary antibody was omitted from the procedure. Images were captured using CellSens imaging software (Olympus Scientific Solutions, Waltham, MA).

For placenta and uterine IF, slides were baked for one hour at 56°C, deparaffinized, and HIER was performed in the Retriever 2100 with R-Universal Epitope Recovery Buffer (62719–10 lot 180314). After retrieval, slides were blocked in 5% normal donkey serum for 1 hour, then primary antibodies in 0.5% normal serum were added and incubated overnight, humidified, at 4°C [MAC-387, macrophage antibody (Abcam, MA); Pan anti-flavivirus; (Millipore, CA)]. The next morning slides were removed from 4°C and allowed to equilibrate to RT, covered, on the benchtop for 1 hour. Slides were rinsed 4 x 5 minutes with PBS, then secondary antibodies were added and incubated 1 hour, covered, at RT. Donkey anti-mouse IgG F(ab’)2 AlexaFluor 594 (Jackson Immunolabs) was used as secondary antibody. Slides were rinsed in PBS, counterstained 5 minutes with DAPI in PBS and cover slipped using Shur/Mount. Cover glass was sealed with nail polish and slides were stored at 4°C and visualized using a fluorescent microscope (Olympus BX43). Images were captured using CellSens imaging software (Olympus).

### Image analysis

Image analysis was performed using NIH ImageJ FIJI (Wayne Rasband, NIH). Original IF images were split into separate channels (Red, green, blue) using identical parameters for each section (minimum, maximum, brightness, contrast). Using LUT, immunostained cells were given a different color to separate from DAPI channel. After the images were restacked to final composite image, using cell counter plugin, cells were counted manually, and then total cells for each section were counted. For counts per animal, average of total cell counts from four sections per animal was used for statistical analysis. For Nestin and DCX stained cells in the DG of the hippocampus, individual cell along the DG was not clearly separated for individual quantification. However, the primary processes from these cells could be properly identified and therefore, were used for quantitation. For Nestin positive cells, NeuronJ plugin in FIJI was used to trace the neurites and the sum of length of all tracings for each section was measured. For DCX+ cell analysis, images were converted to Binary and then skeletonized. 5 fields along the DG were then selected and mean gray value (MGV) of these regions of interest (ROI) and the background was measured. Specific MGV was calculated as the difference between the background MGV and that of each ROI on DG. MGV is a measure of staining intensity in an area, in this case, directly corresponding to DCX+ cell staining along the DG of the hippocampus. For statistical analysis, comparisons between groups were performed using unpaired Student’s *t*-test or one-way ANOVA for groups of three or more, followed by Dunnett’s post-test for multiple comparisons. A p value cut-off of 0.05 was considered statistically significant. All statistical analysis was done using GraphPad Prism software (GraphPad, San Diego, CA).

## References

[1] Dick GW. Zika virus. II. Pathogenicity and physical properties. Transactions of the Royal Society of Tropical Medicine and Hygiene. 1952;46(5):521–34. Epub 1952/09/01. doi: 10.1016/0035-9203(52)90043-6 . 12995441

[2] Dick GW, Kitchen SF, Haddow AJ. Zika virus. I. Isolations and serological specificity. Transactions of the Royal Society of Tropical Medicine and Hygiene. 1952;46(5):509–20. Epub 1952/09/01. doi: 10.1016/0035-9203(52)90042-4 . 12995440

[3] Brasil P, Pereira JP Jr., Moreira ME, Ribeiro Nogueira RM, Damasceno L, Wakimoto M, et al. Zika Virus Infection in Pregnant Women in Rio de Janeiro. N Engl J Med. 2016;375(24):2321–34. doi: 10.1056/NEJMoa1602412 ; PubMed Central PMCID: PMC5323261. 26943629 PMC5323261

[4] Chan JF, Choi GK, Yip CC, Cheng VC, Yuen KY. Zika fever and congenital Zika syndrome: An unexpected emerging arboviral disease. J Infect. 2016;72(5):507–24. Epub 2016/03/05. doi: 10.1016/j.jinf.2016.02.011 . 26940504 PMC7112603

[5] Musso D, Ko AI, Baud D. Zika Virus Infection—After the Pandemic. New England Journal of Medicine. 2019;381(15):1444–57. doi: 10.1056/NEJMra1808246 . 31597021

[6] Reynolds MR, Jones AM, Petersen EE, Lee EH, Rice ME, Bingham A, et al. Vital Signs: Update on Zika Virus-Associated Birth Defects and Evaluation of All U.S. Infants with Congenital Zika Virus Exposure—U.S. Zika Pregnancy Registry, 2016. MMWR Morbidity and mortality weekly report. 2017;66(13):366–73. Epub 2017/04/07. doi: 10.15585/mmwr.mm6613e1 ; PubMed Central PMCID: PMC5657905. 28384133 PMC5657905

[7] Wheeler AC, Toth D, Ridenour T, Lima Nóbrega L, Borba Firmino R, Marques da Silva C, et al. Developmental Outcomes Among Young Children With Congenital Zika Syndrome in Brazil. JAMA network open. 2020;3(5):e204096. Epub 2020/05/06. doi: 10.1001/jamanetworkopen.2020.4096 ; PubMed Central PMCID: PMC7201309 and Roche Therapeutics outside the submitted work. Dr Bailey reported receiving reagents and equipment from Asuragen outside the submitted work. No other disclosures were reported. 32369180 PMC7201309

[8] Vouga M, Pomar L, Panchaud A, Musso D, Baud D. A critical analysis of the neurodevelopmental and neurosensory outcomes after 2 years for children with in utero Zika virus exposure. Nature Medicine. 2019;25(11):1641–2. doi: 10.1038/s41591-019-0630-0 31649352

[9] Masmejan S, Musso D, Vouga M, Pomar L, Dashraath P, Stojanov M, et al. Zika Virus. Pathogens. 2020;9(11). doi: 10.3390/pathogens9110898 ; PubMed Central PMCID: PMC7692141. 33126413 PMC7692141

[10] Einspieler C, Utsch F, Brasil P, Panvequio Aizawa CY, Peyton C, Hydee Hasue R, et al. Association of Infants Exposed to Prenatal Zika Virus Infection With Their Clinical, Neurologic, and Developmental Status Evaluated via the General Movement Assessment Tool. JAMA network open. 2019;2(1):e187235–e. doi: 10.1001/jamanetworkopen.2018.7235 30657537 PMC6431234

[11] van der Linden V, Pessoa A, Dobyns W, Barkovich AJ, Júnior HV, Filho EL, et al. Description of 13 Infants Born During October 2015-January 2016 With Congenital Zika Virus Infection Without Microcephaly at Birth—Brazil. MMWR Morbidity and mortality weekly report. 2016;65(47):1343–8. Epub 2016/12/03. doi: 10.15585/mmwr.mm6547e2 . 27906905

[12] Schuler-Faccini L, Del Campo M, García-Alix A, Ventura LO, Boquett JA, van der Linden V, et al. Neurodevelopment in Children Exposed to Zika in utero: Clinical and Molecular Aspects. Front Genet. 2022;13:758715. Epub 20220308. doi: 10.3389/fgene.2022.758715 ; PubMed Central PMCID: PMC8957982. 35350244 PMC8957982

[13] Rice ME, Galang RR, Roth NM, Ellington SR, Moore CA, Valencia-Prado M, et al. Vital Signs: Zika-Associated Birth Defects and Neurodevelopmental Abnormalities Possibly Associated with Congenital Zika Virus Infection—U.S. Territories and Freely Associated States, 2018. MMWR Morbidity and mortality weekly report. 2018;67(31):858–67. Epub 2018/08/10. doi: 10.15585/mmwr.mm6731e1 ; PubMed Central PMCID: PMC6089332. 30091967 PMC6089332

[14] Vermillion MS, Lei J, Shabi Y, Baxter VK, Crilly NP, McLane M, et al. Intrauterine Zika virus infection of pregnant immunocompetent mice models transplacental transmission and adverse perinatal outcomes. Nat Commun. 2017;8:14575. Epub 2017/02/22. doi: 10.1038/ncomms14575 ; PubMed Central PMCID: PMC5321801. 28220786 PMC5321801

[15] Manangeeswaran M, Ireland DD, Verthelyi D. Zika (PRVABC59) Infection Is Associated with T cell Infiltration and Neurodegeneration in CNS of Immunocompetent Neonatal C57Bl/6 Mice. PLoS Pathog. 2016;12(11):e1006004. Epub 2016/11/18. doi: 10.1371/journal.ppat.1006004 ; PubMed Central PMCID: PMC5113993. 27855206 PMC5113993

[16] Shao Q, Herrlinger S, Yang SL, Lai F, Moore JM, Brindley MA, et al. Zika virus infection disrupts neurovascular development and results in postnatal microcephaly with brain damage. Development. 2016;143(22):4127–36. Epub 2016/11/02. doi: 10.1242/dev.143768 ; PubMed Central PMCID: PMC5117220. 27729407 PMC5117220

[17] Huang WC, Abraham R, Shim BS, Choe H, Page DT. Zika virus infection during the period of maximal brain growth causes microcephaly and corticospinal neuron apoptosis in wild type mice. Scientific reports. 2016;6:34793. Epub 2016/10/08. doi: 10.1038/srep34793 ; PubMed Central PMCID: PMC5054421. 27713505 PMC5054421

[18] Tongthainan D, Mongkol N, Jiamsomboon K, Suthisawat S, Sanyathitiseree P, Sukmak M, et al. Seroprevalence of Dengue, Zika, and Chikungunya Viruses in Wild Monkeys in Thailand. The American journal of tropical medicine and hygiene. 2020;103(3):1228–33. Epub 2020/06/27. doi: 10.4269/ajtmh.20-0057 ; PubMed Central PMCID: PMC7470562. 32588813 PMC7470562

[19] Buechler CR, Bailey AL, Weiler AM, Barry GL, Breitbach ME, Stewart LM, et al. Seroprevalence of Zika Virus in Wild African Green Monkeys and Baboons. mSphere. 2017;2(2). Epub 2017/03/16. doi: 10.1128/mSphere.00392-16 ; PubMed Central PMCID: PMC5343173. 28289727 PMC5343173

[20] Gurung S, Preno AN, Dubaut JP, Nadeau H, Hyatt K, Reuter N, et al. Translational Model of Zika Virus Disease in Baboons. Journal of virology. 2018;92(16). Epub 2018/06/08. doi: 10.1128/JVI.00186-18 ; PubMed Central PMCID: PMC6069201. 29875247 PMC6069201

[21] Gurung S, Reuter N, Preno A, Dubaut J, Nadeau H, Hyatt K, et al. Zika virus infection at mid-gestation results in fetal cerebral cortical injury and fetal death in the olive baboon. PLoS Pathog. 2019;15(1):e1007507. doi: 10.1371/journal.ppat.1007507 ; PubMed Central PMCID: PMC6355048. 30657788 PMC6355048

[22] Peregrine J, Gurung S, Lindgren MC, Husain S, Zavy MT, Myers DA, et al. Zika Virus Infection, Reproductive Organ Targeting, and Semen Transmission in the Male Olive Baboon. Journal of virology. 2019;94(1). Epub 2019/10/11. doi: 10.1128/jvi.01434-19 ; PubMed Central PMCID: PMC6912120. 31597777 PMC6912120

[23] Gurung S, Nadeau H, Maxted M, Peregrine J, Reuter D, Norris A, et al. Maternal Zika Virus (ZIKV) Infection following Vaginal Inoculation with ZIKV-Infected Semen in Timed-Pregnant Olive Baboons. Journal of virology. 2020;94(11). doi: 10.1128/JVI.00058-20 ; PubMed Central PMCID: PMC7269433. 32188737 PMC7269433

[24] Dudley DM, Aliota MT, Mohr EL, Weiler AM, Lehrer-Brey G, Weisgrau KL, et al. A rhesus macaque model of Asian-lineage Zika virus infection. Nat Commun. 2016;7:12204. Epub 2016/06/29. doi: 10.1038/ncomms12204 ; PubMed Central PMCID: PMC4931337. 27352279 PMC4931337

[25] Nguyen SM, Antony KM, Dudley DM, Kohn S, Simmons HA, Wolfe B, et al. Highly efficient maternal-fetal Zika virus transmission in pregnant rhesus macaques. PLoS Pathog. 2017;13(5):e1006378. doi: 10.1371/journal.ppat.1006378 ; PubMed Central PMCID: PMC5444831. 28542585 PMC5444831

[26] Hirsch AJ, Roberts VHJ, Grigsby PL, Haese N, Schabel MC, Wang X, et al. Zika virus infection in pregnant rhesus macaques causes placental dysfunction and immunopathology. Nat Commun. 2018;9(1):263. Epub 2018/01/19. doi: 10.1038/s41467-017-02499-9 ; PubMed Central PMCID: PMC5772047. 29343712 PMC5772047

[27] Coffey LL, Keesler RI, Pesavento PA, Woolard K, Singapuri A, Watanabe J, et al. Intraamniotic Zika virus inoculation of pregnant rhesus macaques produces fetal neurologic disease. Nat Commun. 2018;9(1):2414. doi: 10.1038/s41467-018-04777-6 ; PubMed Central PMCID: PMC6010452. 29925843 PMC6010452

[28] Martinot AJ, Abbink P, Afacan O, Prohl AK, Bronson R, Hecht JL, et al. Fetal Neuropathology in Zika Virus-Infected Pregnant Female Rhesus Monkeys. Cell. 2018;173(5):1111–22 e10. doi: 10.1016/j.cell.2018.03.019 ; PubMed Central PMCID: PMC5959775. 29606355 PMC5959775

[29] Magnani DM, Rogers TF, Maness NJ, Grubaugh ND, Beutler N, Bailey VK, et al. Fetal demise and failed antibody therapy during Zika virus infection of pregnant macaques. Nat Commun. 2018;9(1):1624. doi: 10.1038/s41467-018-04056-4 ; PubMed Central PMCID: PMC5915455. 29691387 PMC5915455

[30] Mohr EL, Block LN, Newman CM, Stewart LM, Koenig M, Semler M, et al. Ocular and uteroplacental pathology in a macaque pregnancy with congenital Zika virus infection. PLoS One. 2018;13(1):e0190617. Epub 2018/01/31. doi: 10.1371/journal.pone.0190617 . 29381706 PMC5790226

[31] Adams Waldorf KM, Stencel-Baerenwald JE, Kapur RP, Studholme C, Boldenow E, Vornhagen J, et al. Fetal brain lesions after subcutaneous inoculation of Zika virus in a pregnant nonhuman primate. Nat Med. 2016;22(11):1256–9. doi: 10.1038/nm.4193 ; PubMed Central PMCID: PMC5365281. 27618651 PMC5365281

[32] Adams Waldorf KM, Nelson BR, Stencel-Baerenwald JE, Studholme C, Kapur RP, Armistead B, et al. Congenital Zika virus infection as a silent pathology with loss of neurogenic output in the fetal brain. Nat Med. 2018;24(3):368–74. doi: 10.1038/nm.4485 ; PubMed Central PMCID: PMC5839998. 29400709 PMC5839998

[33] Koide F, Goebel S, Snyder B, Walters KB, Gast A, Hagelin K, et al. Development of a Zika Virus Infection Model in Cynomolgus Macaques. Front Microbiol. 2016;7:2028. doi: 10.3389/fmicb.2016.02028 ; PubMed Central PMCID: PMC5165249. 28066354 PMC5165249

[34] Haddow AD, Nalca A, Rossi FD, Miller LJ, Wiley MR, Perez-Sautu U, et al. High Infection Rates for Adult Macaques after Intravaginal or Intrarectal Inoculation with Zika Virus. Emerging Infectious Diseases. 2017;23(8):1274–81. doi: 10.3201/eid2308.170036 PMC5547779. 28548637 PMC5547779

[35] Seferovic M, Martin CS, Tardif SD, Rutherford J, Castro ECC, Li T, et al. Experimental Zika Virus Infection in the Pregnant Common Marmoset Induces Spontaneous Fetal Loss and Neurodevelopmental Abnormalities. Scientific reports. 2018;8(1):6851. Epub 2018/05/03. doi: 10.1038/s41598-018-25205-1 ; PubMed Central PMCID: PMC5931554. 29717225 PMC5931554

[36] Berry N, Ferguson D, Ham C, Hall J, Jenkins A, Giles E, et al. High susceptibility, viral dynamics and persistence of South American Zika virus in New World monkey species. Scientific reports. 2019;9(1):14495. Epub 20191010. doi: 10.1038/s41598-019-50918-2 ; PubMed Central PMCID: PMC6787206. 31601848 PMC6787206

[37] Dudley DM, Van Rompay KK, Coffey LL, Ardeshir A, Keesler RI, Bliss-Moreau E, et al. Miscarriage and stillbirth following maternal Zika virus infection in nonhuman primates. Nat Med. 2018;24(8):1104–7. Epub 2018/07/04. doi: 10.1038/s41591-018-0088-5 ; PubMed Central PMCID: PMC6082723. 29967348 PMC6082723

[38] Dudley DM, Aliota MT, Mohr EL, Newman CM, Golos TG, Friedrich TC, et al. Using Macaques to Address Critical Questions in Zika Virus Research. Annu Rev Virol. 2019;6(1):481–500. doi: 10.1146/annurev-virology-092818-015732 ; PubMed Central PMCID: PMC7323203. 31180813 PMC7323203

[39] Narasimhan H, Chudnovets A, Burd I, Pekosz A, Klein SL. Animal models of congenital zika syndrome provide mechanistic insight into viral pathogenesis during pregnancy. PLoS Negl Trop Dis. 2020;14(10):e0008707. doi: 10.1371/journal.pntd.0008707 ; PubMed Central PMCID: PMC7580937. 33091001 PMC7580937

[40] Van Rompay KKA, Keesler RI, Ardeshir A, Watanabe J, Usachenko J, Singapuri A, et al. DNA vaccination before conception protects Zika virus-exposed pregnant macaques against prolonged viremia and improves fetal outcomes. Sci Transl Med. 2019;11(523). doi: 10.1126/scitranslmed.aay2736 ; PubMed Central PMCID: PMC7093037. 31852797 PMC7093037

[41] Steinbach RJ, Haese NN, Smith JL, Colgin LMA, MacAllister RP, Greene JM, et al. A neonatal nonhuman primate model of gestational Zika virus infection with evidence of microencephaly, seizures and cardiomyopathy. PLoS One. 2020;15(1):e0227676. Epub 20200114. doi: 10.1371/journal.pone.0227676 ; PubMed Central PMCID: PMC6959612. 31935257 PMC6959612

[42] Crooks CM, Weiler AM, Rybarczyk SL, Bliss M, Jaeger AS, Murphy ME, et al. African-Lineage Zika Virus Replication Dynamics and Maternal-Fetal Interface Infection in Pregnant Rhesus Macaques. Journal of virology. 2021;95(16):e0222020. Epub 20210726. doi: 10.1128/JVI.02220-20 ; PubMed Central PMCID: PMC8312872. 34076485 PMC8312872

[43] Koenig MR, Razo E, Mitzey A, Newman CM, Dudley DM, Breitbach ME, et al. Quantitative definition of neurobehavior, vision, hearing and brain volumes in macaques congenitally exposed to Zika virus. PLoS One. 2020;15(10):e0235877. Epub 20201022. doi: 10.1371/journal.pone.0235877 ; PubMed Central PMCID: PMC7580995. 33091010 PMC7580995

[44] Nogueira ML, Nery Júnior NRR, Estofolete CF, Bernardes Terzian AC, Guimarães GF, Zini N, et al. Adverse birth outcomes associated with Zika virus exposure during pregnancy in São José do Rio Preto, Brazil. Clin Microbiol Infect. 2018;24(6):646–52. Epub 20171110. doi: 10.1016/j.cmi.2017.11.004 . 29133154

[45] Roth NM, Reynolds MR, Lewis EL, Woodworth KR, Godfred-Cato S, Delaney A, et al. Zika-Associated Birth Defects Reported in Pregnancies with Laboratory Evidence of Confirmed or Possible Zika Virus Infection—U.S. Zika Pregnancy and Infant Registry, December 1, 2015-March 31, 2018. MMWR Morbidity and mortality weekly report. 2022;71(3):73–9. Epub 20220121. doi: 10.15585/mmwr.mm7103a1 ; PubMed Central PMCID: PMC8774158. 35051132 PMC8774158

[46] Shearer MH, Dark RD, Chodosh J, Kennedy RC. Comparison and characterization of immunoglobulin G subclasses among primate species. Clin Diagn Lab Immunol. 1999;6(6):953–8. doi: 10.1128/CDLI.6.6.953-958.1999 ; PubMed Central PMCID: PMC95804. 10548592 PMC95804

[47] Shearer MH, Lucas AH, Anderson PW, Carey KD, Jenson HB, Chanh TC, et al. The baboon as a nonhuman primate model for assessing the effects of maternal immunization with Haemophilus influenzae type b polysaccharide vaccines. Infect Immun. 1997;65(8):3267–70. doi: 10.1128/iai.65.8.3267-3270.1997 ; PubMed Central PMCID: PMC175462. 9234785 PMC175462

[48] Wolf RF, Papin JF, Hines-Boykin R, Chavez-Suarez M, White GL, Sakalian M, et al. Baboon model for West Nile Virus infection and vaccine evaluation. Virology. 2006;355(1):44–51. doi: 10.1016/j.virol.2006.06.033 16904151

[49] Gurung S, Preno AN, Dubaut JP, Nadeau H, Hyatt K, Reuter N, et al. Translational Model of Zika Virus Disease in Baboons. J Virol. 2018. Epub 2018/06/08. doi: 10.1128/JVI.00186-18 ; PubMed Central PMCID: PMC6069201. 29875247 PMC6069201

[50] Kochunov P, Castro C, Davis D, Dudley D, Brewer J, Zhang Y, et al. Mapping primary gyrogenesis during fetal development in primate brains: high-resolution in utero structural MRI of fetal brain development in pregnant baboons. Front Neurosci. 2010;4:20. doi: 10.3389/fnins.2010.00020 ; PubMed Central PMCID: PMC2896074. 20631812 PMC2896074

[51] Krieg WJ. Connections of the cerebral cortex; the macaque; topography. J Comp Neurol. 1949;91(1):1–38, illust. doi: 10.1002/cne.900910102 . 15391841

[52] de Abreu T, Tavares MCH, Bretas R, Rodrigues RC, Pissinati A, Aversi-Ferreira TA. Comparative anatomy of the encephalon of new world primates with emphasis for the Sapajus sp. PLoS One. 2021;16(9):e0256309. doi: 10.1371/journal.pone.0256309 ; PubMed Central PMCID: PMC8409804. 34469439 PMC8409804

[53] Mikula S, Trotts I, Stone JM, Jones EG. Internet-enabled high-resolution brain mapping and virtual microscopy. Neuroimage. 2007;35(1):9–15. doi: 10.1016/j.neuroimage.2006.11.053 ; PubMed Central PMCID: PMC1890021. 17229579 PMC1890021

[54] Pekny M, Pekna M. Reactive gliosis in the pathogenesis of CNS diseases. Biochim Biophys Acta. 2016;1862(3):483–91. Epub 20151202. doi: 10.1016/j.bbadis.2015.11.014 . 26655603

[55] Panganiban AT, Blair RV, Hattler JB, Bohannon DG, Bonaldo MC, Schouest B, et al. A Zika virus primary isolate induces neuroinflammation, compromises the blood-brain barrier and upregulates CXCL12 in adult macaques. Brain Pathol. 2020;30(6):1017–27. Epub 20200721. doi: 10.1111/bpa.12873 ; PubMed Central PMCID: PMC8018016. 32585067 PMC8018016

[56] Lozier MJ, Rosenberg ES, Doyle K, Adams L, Klein L, Munoz-Jordan J, et al. Prolonged detection of Zika virus nucleic acid among symptomatic pregnant women: a cohort study. Clin Infect Dis. 2018. Epub 2018/03/14. doi: 10.1093/cid/ciy209 . 29534160 PMC6927853

[57] Singh T, Lopez CA, Giuberti C, Dennis ML, Itell HL, Heimsath HJ, et al. Efficient transplacental IgG transfer in women infected with Zika virus during pregnancy. PLoS Negl Trop Dis. 2019;13(8):e0007648. Epub 20190826. doi: 10.1371/journal.pntd.0007648 ; PubMed Central PMCID: PMC6730934. 31449521 PMC6730934

[58] Driggers RW, Ho CY, Korhonen EM, Kuivanen S, Jääskeläinen AJ, Smura T, et al. Zika Virus Infection with Prolonged Maternal Viremia and Fetal Brain Abnormalities. N Engl J Med. 2016;374(22):2142–51. Epub 20160330. doi: 10.1056/NEJMoa1601824 . 27028667

[59] Suy A, Sulleiro E, Rodó C, Vázquez É, Bocanegra C, Molina I, et al. Prolonged Zika Virus Viremia during Pregnancy. N Engl J Med. 2016;375(26):2611–3. Epub 20161207. doi: 10.1056/NEJMc1607580 . 27959695

[60] Pillay P, Manger PR. Order-specific quantitative patterns of cortical gyrification. The European journal of neuroscience. 2007;25(9):2705–12. Epub 2007/04/27. doi: 10.1111/j.1460-9568.2007.05524.x . 17459107

[61] Leigh SR. Brain growth, life history, and cognition in primate and human evolution. American journal of primatology. 2004;62(3):139–64. Epub 2004/03/18. doi: 10.1002/ajp.20012 . 15027089

[62] Kochunov P, Duff Davis M. Development of structural MR brain imaging protocols to study genetics and maturation. Methods (San Diego, Calif). 2010;50(3):136–46. Epub 2009/08/12. doi: 10.1016/j.ymeth.2009.08.002 ; PubMed Central PMCID: PMC2828529. 19665566 PMC2828529

[63] Stiles J, Jernigan TL. The basics of brain development. Neuropsychol Rev. 2010;20(4):327–48. doi: 10.1007/s11065-010-9148-4 ; PubMed Central PMCID: PMC2989000. 21042938 PMC2989000

[64] Hazin AN, Poretti A, Di Cavalcanti Souza Cruz D, Tenorio M, van der Linden A, Pena LJ, et al. Computed Tomographic Findings in Microcephaly Associated with Zika Virus. N Engl J Med. 2016;374(22):2193–5. Epub 2016/04/07. doi: 10.1056/NEJMc1603617 . 27050112

[65] Mlakar J, Korva M, Tul N, Popović M, Poljšak-Prijatelj M, Mraz J, et al. Zika Virus Associated with Microcephaly. N Engl J Med. 2016;374(10):951–8. Epub 2016/02/11. doi: 10.1056/NEJMoa1600651 . 26862926

[66] Stahl R, Walcher T, De Juan Romero C, Pilz GA, Cappello S, Irmler M, et al. Trnp1 regulates expansion and folding of the mammalian cerebral cortex by control of radial glial fate. Cell. 2013;153(3):535–49. Epub 2013/04/30. doi: 10.1016/j.cell.2013.03.027 . 23622239

[67] Sun T, Hevner RF. Growth and folding of the mammalian cerebral cortex: from molecules to malformations. Nat Rev Neurosci. 2014;15(4):217–32. doi: 10.1038/nrn3707 ; PubMed Central PMCID: PMC4107216. 24646670 PMC4107216

[68] Schaer M, Ottet M-C, Scariati E, Dukes D, Franchini M, Eliez S, et al. Decreased frontal gyrification correlates with altered connectivity in children with autism. Frontiers in Human Neuroscience. 2013;7(750). doi: 10.3389/fnhum.2013.00750 24265612 PMC3820980

[69] Palaniyappan L, Liddle PF. Differential effects of surface area, gyrification and cortical thickness on voxel based morphometric deficits in schizophrenia. Neuroimage. 2012;60(1):693–9. Epub 2012/01/10. doi: 10.1016/j.neuroimage.2011.12.058 . 22227049

[70] Waxman SG. Clinical Neuroanatomy 26 ed: McGraw-Hill; 2010.

[71] Soares de Oliveira-Szejnfeld P, Levine D, Melo AS, Amorim MM, Batista AG, Chimelli L, et al. Congenital Brain Abnormalities and Zika Virus: What the Radiologist Can Expect to See Prenatally and Postnatally. Radiology. 2016;281(1):203–18. Epub 2016/08/24. doi: 10.1148/radiol.2016161584 . 27552432

[72] de Fatima Vasco Aragao M, van der Linden V, Brainer-Lima AM, Coeli RR, Rocha MA, Sobral da Silva P, et al. Clinical features and neuroimaging (CT and MRI) findings in presumed Zika virus related congenital infection and microcephaly: retrospective case series study. BMJ (Clinical research ed). 2016;353:i1901. Epub 2016/04/15. doi: 10.1136/bmj.i1901 ; PubMed Central PMCID: PMC4830901 www.icmje.org/coi_disclosure.pdf and declare: no support from any organisation for the submitted work; no financial relationships with any organisations that might have an interest in the submitted work in the previous three years; no other relationships or activities that could appear to have influenced the submitted work. 27075009 PMC4830901

[73] Schmahmann JD. The cerebellum and cognition. Neurosci Lett. 2019;688:62–75. doi: 10.1016/j.neulet.2018.07.005 . 29997061

[74] Strick PL, Dum RP, Fiez JA. Cerebellum and Nonmotor Function. Annual Review of Neuroscience. 2009;32(1):413–34. doi: 10.1146/annurev.neuro.31.060407.125606 . 19555291

[75] Vhp L, Aragao MM, Pinho RS, Hazin AN, Paciorkowski AR, Penalva de Oliveira AC, et al. Congenital Zika Virus Infection: a Review with Emphasis on the Spectrum of Brain Abnormalities. Curr Neurol Neurosci Rep. 2020;20(11):49. doi: 10.1007/s11910-020-01072-0 ; PubMed Central PMCID: PMC7468090. 32880775 PMC7468090

[76] Cantelmi D, Schweizer TA, Cusimano MD. Role of the cerebellum in the neurocognitive sequelae of treatment of tumours of the posterior fossa: an update. The Lancet Oncology. 2008;9(6):569–76. Epub 2008/05/31. doi: 10.1016/S1470-2045(08)70148-7 . 18510988

[77] Fernández-Arjona MdM Grondona JM, Granados-Durán P, Fernández-Llebrez P, López-Ávalos MD. Microglia Morphological Categorization in a Rat Model of Neuroinflammation by Hierarchical Cluster and Principal Components Analysis. Frontiers in Cellular Neuroscience. 2017;11(235). doi: 10.3389/fncel.2017.00235 28848398 PMC5550745

[78] de Sousa JR, Azevedo RSS, Martins Filho AJ, Araujo MTF, Moutinho ERC, Baldez Vasconcelos BC, et al. Correlation between Apoptosis and in Situ Immune Response in Fatal Cases of Microcephaly Caused by Zika Virus. The American journal of pathology. 2018;188(11):2644–52. Epub 2018/08/20. doi: 10.1016/j.ajpath.2018.07.009 . 30121258

[79] Potokar M, Jorgačevski J, Zorec R. Astrocytes in Flavivirus Infections. International journal of molecular sciences. 2019;20(3). Epub 2019/02/10. doi: 10.3390/ijms20030691 ; PubMed Central PMCID: PMC6386967. 30736273 PMC6386967

[80] Pomar L, Lambert V, Madec Y, Vouga M, Pomar C, Matheus S, et al. Placental infection by Zika virus in French Guiana. Ultrasound in obstetrics & gynecology: the official journal of the International Society of Ultrasound in Obstetrics and Gynecology. 2020;56(5):740–8. Epub 2019/11/28. doi: 10.1002/uog.21936 . 31773804

[81] Santos GR, Pinto CAL, Prudente RCS, Bevilacqua E, Witkin SS, Passos SD. Histopathologic Changes in Placental Tissue Associated With Vertical Transmission of Zika Virus. International journal of gynecological pathology: official journal of the International Society of Gynecological Pathologists. 2020;39(2):157–62. Epub 2019/02/23. doi: 10.1097/PGP.0000000000000586 . 30789499

[82] Styer LM, Kent KA, Albright RG, Bennett CJ, Kramer LD, Bernard KA. Mosquitoes inoculate high doses of West Nile virus as they probe and feed on live hosts. PLoS Pathog. 2007;3(9):1262–70. doi: 10.1371/journal.ppat.0030132 ; PubMed Central PMCID: PMC1976553. 17941708 PMC1976553

[83] Dutra HL, Rocha MN, Dias FB, Mansur SB, Caragata EP, Moreira LA. Wolbachia Blocks Currently Circulating Zika Virus Isolates in Brazilian Aedes aegypti Mosquitoes. Cell Host Microbe. 2016;19(6):771–4. doi: 10.1016/j.chom.2016.04.021 ; PubMed Central PMCID: PMC4906366. 27156023 PMC4906366

[84] Dudley DM, Newman CM, Lalli J, Stewart LM, Koenig MR, Weiler AM, et al. Infection via mosquito bite alters Zika virus tissue tropism and replication kinetics in rhesus macaques. Nat Commun. 2017;8(1):2096. Epub 2017/12/14. doi: 10.1038/s41467-017-02222-8 ; PubMed Central PMCID: PMC5727388. 29235456 PMC5727388

[85] Lanciotti RS, Kosoy OL, Laven JJ, Velez JO, Lambert AJ, Johnson AJ, et al. Genetic and serologic properties of Zika virus associated with an epidemic, Yap State, Micronesia, 2007. Emerg Infect Dis. 2008;14(8):1232–9. doi: 10.3201/eid1408.080287 ; PubMed Central PMCID: PMC2600394. 18680646 PMC2600394

